# The Foxp3^+^ regulatory T-cell population requires IL-4Rα signaling to control inflammation during helminth infections

**DOI:** 10.1371/journal.pbio.2005850

**Published:** 2018-10-31

**Authors:** Nada Abdel Aziz, Justin Komguep Nono, Thabo Mpotje, Frank Brombacher

**Affiliations:** 1 Cytokines and Diseases Group, International Centre for Genetic Engineering and Biotechnology, Cape Town Component, Cape Town, South Africa; 2 University of Cape Town, Institute of Infectious Diseases and Molecular Medicine (IDM), Department of Pathology, Division of Immunology and South African Medical Research Council (SAMRC) Immunology of Infectious Diseases, Faculty of Health Sciences, University of Cape Town, Cape Town, South Africa; 3 Biotechnology/Biomolecular Chemistry Program, Chemistry Department, Faculty of Science, Cairo University, Cairo, Egypt; 4 The Medical Research Centre, Institute of Medical Research and Medicinal Plant Studies (IMPM), Ministry of Scientific Research and Innovation, Yaoundé, Cameroon; 5 Wellcome Centre for Infectious Diseases Research in Africa, Institute of Infectious Diseases and Molecular Medicine (IDM), Faculty of Health Sciences, University of Cape Town, Cape Town, South Africa; National Jewish Health, United States of America

## Abstract

Forkhead box P3 (Foxp3^+^) regulatory T (Treg)-cell function is controlled by environmental cues of which cytokine-mediated signaling is a dominant component. In vivo, interleukin-4 (IL-4)-mediated signaling via IL-4 receptor alpha (IL-4Rα) mediates Treg cell transdifferentiation into ex-Foxp3 T helper 2 (Th2) or T helper 17 (Th17) cells. However, IL-4-mediated signaling also reinforces the Foxp3 Treg compartment in vitro. We generated Foxp3-specific IL-4Rα-deficient mice and demonstrated differential efficiency of IL-4Rα deletion in male (approximately 90%) and female (approximately 40%) animals, because of cyclic recombinase (Cre)-mediated X-linked *foxp3* inactivation. Irrespective of the degree of IL-4Rα deletion within the Foxp3^+^ Treg cell population, mice showed exacerbation of immune effector responses with aggravated tissue pathology in tissue-dwelling helminth infections (*Schistosoma mansoni* or *Nippostrongylus brasiliensis*). Mechanistically, IL-4Rα deletion in males and females led to a reduced expression of Foxp3 and subsequently an impaired accumulation of Foxp3^+^ Treg cells to inflamed tissues. In-depth cellular typing by flow cytometry revealed that the impairment of IL-4Rα-mediated signaling during helminth infections decreased the ability of central Treg cells to convert into effector Treg (eTreg) cells and caused a significant down-regulation of markers associated with Treg cell migration (C-X-C motif chemokine receptor 3 [CXCR3]) and accumulation in inflamed tissues (GATA binding protein 3 [GATA3]) as well as survival (B cell lymphoma 2 [Bcl-2]). These findings unprecedentedly, to our knowledge, uncover a role for IL-4Rα signaling in the positive regulation of Foxp3^+^ Treg cell function in vivo. Complementing our past knowledge on a widely reported role for IL-4Rα signaling in the negative regulation and transdifferentiation of Foxp3^+^ Treg cells in vivo, our present findings reveal the host requirement for an intact, but not reduced or potentiated, IL-4Rα-mediated signaling on Foxp3^+^ Treg cells to optimally control inflammation during helminth infections.

## Author summary

Host soluble mediators such as cytokines play a key role in the regulation of the immune response. Forkhead box P3 (Foxp3^+^) regulatory T (Treg) cells, which are involved in maintaining self-tolerance and immune system homeostasis, are influenced by cytokines, including interleukin-4 (IL-4). However, opposing reports have emerged on the effect of this cytokine on Treg cells. Some evidence suggests IL-4 inhibits Treg cells, whereas other studies indicate a supportive role for this cytokine in Treg cell biology and function. To unambiguously address this question, we generated mice with IL-4 receptor specifically removed from the Treg cell population. Our newly generated mice did not show any sign of spontaneous inflammation during homeostasis, but when challenged with an experimental infection by parasitic worms, deletion of the IL-4 receptor from the Treg cell population led to increased inflammation and aggravated tissue pathology. Several defects such as poor activation, reduced promigratory marker expression, and reduced survival were apparent in Treg cells with impaired IL-4 responsiveness. Our evidence presents a strong case for a supportive role of IL-4 via IL-4 receptor in the biology and optimal regulatory function of Treg cells during worm infections.

## Introduction

Regulatory T (Treg) cells, the central component in the regulation of the immune system, play a pivotal role in the maintenance of self-tolerance and immune homeostasis [1]. Research on the molecular bases of Treg cells’ function has revealed the X-linked transcription factor forkhead box P3 (Foxp3) as uniquely expressed in Treg cells. How Foxp3^+^ Treg cells are regulated is a critical question yet to be fully explored. Growing evidence has established that Foxp3^+^ Treg cell function is greatly affected by the integrity of their receptors and the cytokines available in the milieu. Our knowledge of the intricacies of such regulation has now considerably expanded.

Cytokines such as interleukin-2 (IL-2), interleukin-15 (IL-15) [2–4], interferon gamma (IFN-γ) [5,6], interleukin-12 (IL-12) [7], interleukin-6 (IL-6) [8,9], and interleukin-4 (IL-4) [10–15] have been reported to provide critical signals in the modulation of Treg cells’ development and function. IL-4, the canonical cytokine defining Type 2 immune responses, signals through the IL-4 receptor alpha (IL-4Rα) to mitigate Treg cell function during Type 2 diseases [16,17]. Recent reports have shown that augmentation of IL-4Rα signaling through gain-of-function mutation [14,15] or chronic Type 2 inflammation [18] leads to a drastic reduction in Foxp3^+^ Treg cell population and impairment of Treg cell suppressive function, which in turn drive their reprogramming toward T helper 2 (Th2)-like or T helper 17 (Th17)-like cells [14,15,18], favoring the notion of an inhibitory role for this receptor in Treg cell function. However, treatment of cluster of differentiation 4 (CD4)^+^ cluster of differentiation 25 (CD25)^+^ Treg cells in vitro with IL-4 has been shown to have an antiapoptotic role, an augmentation of the rate of Foxp3 expression, and potentiation of Foxp3^+^ Treg cell suppressive function [10], suggesting an unappreciated supporting role for IL-4Rα-mediated signaling in Foxp3^+^ Treg cell function in vitro yet to be validated in vivo.

In the present study, we show that Foxp3^+^ Treg cells in secondary lymphoid organs constitutively express IL-4Rα and up-regulate the receptor expression upon *S*. *mansoni* (*Sm*) infection. We provide evidence that either 40% or 90% of deletion of the IL-4 receptor specifically within the Foxp3^+^ Treg cell population leads to aggravated tissue inflammation during helminth infections (*Sm* and *N*. *brasiliensis* [*Nb*] infections) and that this occurs as a result of a weakened Foxp3^+^ Treg cell compartment paralleled by an uncontrolled effector T-cell compartment. Adding to previous reports that have defined a restraining role for IL-4Rα on Foxp3^+^ Treg cells in vivo, we now present complementary evidence indicating that this receptor is equally needed, in full, in vivo for Foxp3^+^ Treg cell ability to control inflammation during helminth infections.

## Results

### Foxp3^+^ Treg cells up-regulate IL-4Rα expression after *Sm* infection

**Fig 1:**
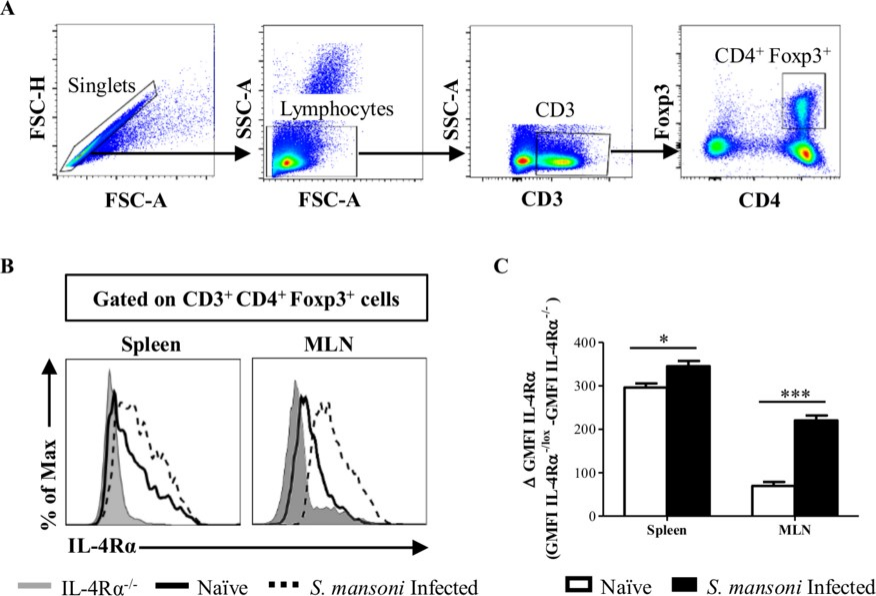
Foxp3^+^ Treg cells up-regulate IL-4Rα expression upon infection. (A) Gating strategy for identifying Foxp3^+^ Treg cell population. (B) Representative histograms of IL-4Rα expression by Foxp3^**+**^ Treg cells in spleen and MLNs of IL-4Rα^**−/−**^ (gray tinted), naïve (solid line), and *Sm*-infected (dashed line) IL-4Rα^**−/**lox^ mice with the mean of Δ GMFI IL-4Rα (GMFI IL-4Rα^**−/**lox^ − GMFI IL-4Rα^−/−^) ± S.E.M summarized in (C). Data are from two independent experiments. *n* = 6–8 mice/group. * *P* < 0.05, ** *P* < 0.001, *** *P* < 0.0001 by two-tailed unpaired Student *t* test. Underlying data can be found in S1 Data. CD3, cluster of differentiation 3; CD4, cluster of differentiation 4; Foxp3, forkhead box P3; FSC-A, forward scatter A; FSC-H, forward scatter H; GMFI, geometric mean fluorescence intensity; IL-4Rα, interleukin-4 receptor alpha; MLN, mesenteric lymph node; *Sm*, *S*. *mansoni*; SSC-A, side scatter A; Treg, regulatory T.

IL-4Rα-mediated signaling and associated factors critically mediating the function of Th2 cells were recently shown to play an important role in controlling Foxp3^+^ Treg cell function [10,12,14,15,18–20]. However, whether IL-4Rα-mediated signaling on Foxp3^+^ Treg cells promotes [10,19] or inhibits [14,15,18] their suppressive function(s) remains unclear. To address this, we examined the surface protein expression pattern of IL-4Rα on Foxp3^+^ Treg cells in spleen and mesenteric lymph nodes (MLNs) of naïve and 8-wk-*Sm*-infected BALB/c mice. CD4^+^ Foxp3^+^ Treg cells expressed IL-4Rα under a steady state and up-regulated their expression upon *Sm* infection (Fig 1A–1C). These results suggest that IL-4Rα-mediated signaling might be important for Foxp3^+^ Treg cells to function under steady-state and inflammatory conditions.

### Generation and characterization of Foxp3^cre^ IL-4Rα^−/lox^ BALB/c mice

**Fig 2:**
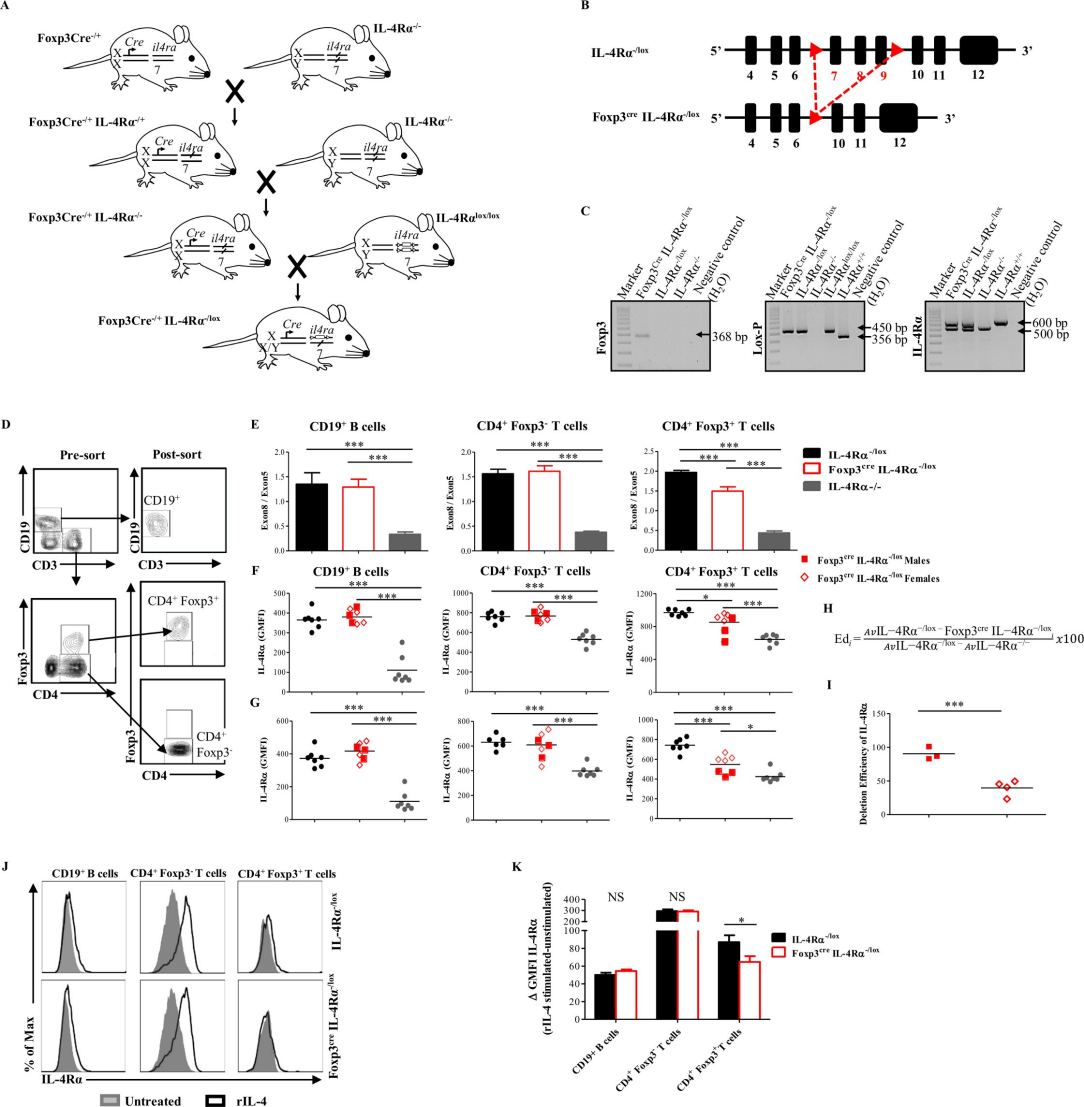
Generation and characterization of Foxp3^Cre−/+^ IL-4Rα^−/lox^ mice. (A) Mouse breeding strategy. IL-4Rα^−/−^ BALB/c mice were intercrossed for two generations with transgenic BALB/c mice expressing Cre-recombinase under control of *foxp3* gene promoter and IL-4Rα^lox/lox^ mice to generate Foxp3Cre^−/+^ IL-4Rα^−/lox^ BALB/c mice. (B) Schematic diagram showing the structure of *il4ra* gene loci in IL-4Rα^−/lox^ and Foxp3^cre^ IL-4Rα^−/lox^ mice. (C) Genotyping of Foxp3Cre^−/+^ IL-4Rα^−/lox^ mice. The Cre specific amplicon is 368 bp, loxP is 450 bp (floxed) or 356 bp (wild type), the wild-type IL4-Rα amplicon is 600 bp, and the deleted IL-4Rα amplicon is 471 bp. (D) Representative flow cytometric analysis of the CD19^+^, CD4^+^ Foxp3^−^, and CD4^+^ Foxp3^+^ cell populations before and after FACS sorting of pooled cells from spleen and MLN of naïve IL-4Rα^−/lox^, Foxp3^cre^ IL-4Rα^−/lox^, and IL-4Rα^−/−^ mice. (E) Efficiency of IL-4Rα deletion by qPCR. Genomic DNA was extracted from sorted CD19^+^ B cells, CD4^+^ Foxp3^−^, and CD4^+^ Foxp3^+^ T cell, and *il4ra* exon 8 (deleted in IL-4Rα deficient cells) was quantified by qPCR and normalized to the quantity of exon 5 (present in all cells). (F) Flow cytometry analysis of IL-4Rα expression by CD19^+^ B cells, CD4^+^ Foxp3^−^, and CD4^+^ Foxp3^+^ T cell from the spleen of naïve mice. (G) Flow cytometry analysis of IL-4Rα expression by CD19^+^ B cells, CD4^+^ Foxp3^−^, and CD4^+^ Foxp3^+^ T cell from the MLNs of naïve mice. (H) Formula for calculating the Ed of *il4ra* gene. (I) Efficiency of IL-4Rα deletion on CD4^+^ Foxp3^+^ T cells in the MLNs of naïve male and female Foxp3^cre^ IL-4Rα^−/lox^ mice. (J) Representative histograms of IL-4Rα expression before (gray tinted) and after recombinant IL-4 (black solid line) stimulation. Cells pooled from spleen and MLNs from naïve IL-4Rα^−/lox^ and Foxp3^cre^ IL-4Rα^−/lox^ mice were cultured for 40 hr in 0 or 1 ng/ml of rIL-4. (K) Flow cytometry analysis of IL-4Rα GMFI up-regulation from (J). Results are representative of three independent experiments with 6–8 mice/group. Data are expressed as mean ± S.E.M. NS, *P* > 0.05; * *P* < 0.05, ** *P* < 0.001, *** *P* < 0.0001 by two-tailed unpaired Student *t* test and one-way ANOVA with Bonferroni posttest analysis. Underlying data can be found in S1 Data. CD3, cluster of differentiation 3; CD4, cluster of differentiation 4; CD19, cluster of differentiation 19; Cre, cyclic recombinase; Ed, efficiency of deletion; FACS, fluorescence-activated cell sorting; Foxp3, forkhead box P3; GMFI, geometric mean fluorescence intensity; IL-4Rα, interleukin-4 receptor alpha; loxP, locus of X-over P1; MLN, mesenteric lymph node; NS, not significant; qPCR, quantitative real-time PCR; rIL-4, recombinant interleukin-4.

To better dissect the role of IL-4Rα-mediated signaling on Foxp3^+^ Treg cells, we generated a murine model, termed Foxp3^cre^ IL-4Rα^−/lox^, with a specific cyclic recombinase (Cre)-mediated deletion of the *il-4rα* gene in Foxp3-expressing cells. Foxp3^cre^ IL-4Rα^−/lox^ mice were generated by intercrossing BALB/c mice expressing Cre under control of *foxp3* gene promoter [21] with global knock-out (IL-4Rα^−/−^) BALB/c mice [22] for two generations to generate Foxp3^cre^ IL-4Rα^−/−^ BALB/c mice (Figs 2A and S1). These mice were further intercrossed with homozygous floxed IL-4Rα (IL-4Rα^lox/lox^) BALB/c mice (exon 7 to 9 flanked by *LoxP* sites; Fig 2B) [23] to generate a Foxp3-specific IL-4Rα-deficient mouse BALB/c strain (Foxp3^cre^ IL-4Rα^−/lox^ BALB/c mice; Figs 2A and S1). Foxp3^cre^ IL-4Rα^−/lox^ mice were identified by PCR genotyping (Fig 2C). The cellular specificity of Cre-mediated IL-4Rα deletion was assessed at the genomic level by performing quantitative real-time PCR (qPCR). Genomic DNA was extracted from cluster of differentiation 19 (CD19)^+^, CD4^+^ Foxp3^−^, and CD4^+^ Foxp3^+^ sorted cells from pooled spleen and MLN cells of naïve Foxp3^cre^ IL-4Rα^−/lox^, their littermate control (IL-4Rα^−/lox^), and global knock-out (IL-4Rα^−/−^) mice (Fig 2D); and *il-4rα* exon 8 (absent in IL-4Rα-deficient cells) was quantified by qPCR and normalized to *il-4rα* exon 5 (present in all cells). As expected, only CD4^+^ Foxp3^+^ T cells, but not CD19^+^ or CD4^+^ Foxp3^−^ cells, from Foxp3^cre^ IL-4Rα^−/lox^ mice had a lower exon8:exon5 ratio when compared to their littermate controls (Fig 2E). Genotypic deletion of *il-4rα* within CD4^+^ Foxp3^+^ Treg cells in Foxp3^cre^ IL-4Rα^−/lox^ mice was further confirmed at the protein level by flow cytometry analyses of IL-4Rα surface expression on spleen and MLN cells of naïve mice. In both organs, IL-4Rα was deleted specifically within CD4^+^ Foxp3^+^ Treg cell population in Foxp3^cre^ IL-4Rα^−/lox^ male and female mice (Fig 2F and 2G). *il-4rα* gene deletion within the CD4^+^ Foxp3^+^ Treg cell population was more efficient in males (efficiency of deletion [Ed] = 90.48% ± 5.45%, Fig 2H and 2I) when compared to females (Ed = 39.74% ± 5.776%, Fig 2H and 2I), enabling via the present model an assessment of the effect of partial (female) versus quasi-complete (male) impairment of IL-4Rα-mediated signaling on CD4^+^ Foxp3^+^ Treg cell in Foxp3^cre^ IL-4Rα^−/lox^ mice.

To assess the functional impairment of IL-4Rα-mediated signaling on CD4^+^ Foxp3^+^ Treg cells in our newly generated model, pooled spleen and MLN cells of naïve male and female Foxp3^cre^ IL-4Rα^−/lox^ mice and their littermate controls were cultured with or without recombinant IL-4 (rIL-4) for 40 hr and/or 1 hr, and then, levels of IL-4Rα surface expression and signal transducer and activator of transcription 6 (STAT6) phosphorylation were measured by flow cytometry, respectively. CD19^+^ B cells and CD4^+^ Foxp3^−^ T cells from Foxp3^cre^ IL-4Rα^−/lox^ mice and their littermate control had a comparable level of IL-4Rα expression, as determined by IL-4Rα geometric mean fluorescence intensity (GMFI), after the addition of rIL-4 (Fig 2J and 2K). In agreement, no major differences in the level of phosphorylated STAT6 (p-STAT6) either at baseline (S2A and S2B Fig) or after rIL-4 stimulation (S2A Fig and S2C–1E) in both populations (CD19^+^ B cells and CD4^+^ Foxp3^−^ T cells) were noted between Foxp3^cre^ IL-4Rα^−/lox^ mice and their littermate controls. In contrast, a proportion of rIL-4-stimulated CD4^+^ Foxp3^+^ Treg cells derived from Foxp3^cre^ IL-4Rα^−/lox^ mice were significantly impaired in their ability to up-regulate IL-4Rα expression compared to CD4^+^ Foxp3^+^ Treg cells derived from their littermate control (Fig 2J and 2K). In support, STAT6 phosphorylation upon rIL-4 stimulation in CD4^+^ Foxp3^+^ Treg cells in IL-4Rα^−/lox^ mice was significantly higher compared to Foxp3^cre^ IL-4Rα^−/lox^ mice (S2A and S2C Fig), indicating that the IL-4Rα signaling pathway on CD4^+^ Foxp3^+^ Treg cells is impaired in our Foxp3^cre^ IL-4Rα^−/lox^ mice. Of interest, we noted that at baseline, male mice do have a higher level of p-STAT6 when compared to female mice (S2B Fig). In our Foxp3^cre^ IL-4Rα^−/lox^ mice, even though the increase in STAT6 phosphorylation upon rIL-4 stimulation in Foxp3^+^ Treg cell compartment was higher in female mice than males (S2D and S2E Fig) because of the differential deletion of *il4ra* gene on Foxp3^+^ Treg cells, the initial higher level of p-STAT6 noticed in male mice brought the absolute final level of p-STAT6 in male and female mice to a comparable level (S2C Fig).

Collectively, these results reveal that IL-4Rα is specifically deleted on CD4^+^ Foxp3^+^ Treg cells in Foxp3^cre^ IL-4Rα^−/lox^ mice either partially (approximately 40% in females) or quasi-completely (approximately 90% in males), resulting—in both cases—in a significant impairment of IL-4Rα-mediated signaling in the Foxp3^+^ Treg cell population.

### Impairment of IL-4Rα within Foxp3^+^ Treg cells neither alters Foxp3^+^ Treg cell compartment nor breaks tolerance under a steady state

**Fig 3:**
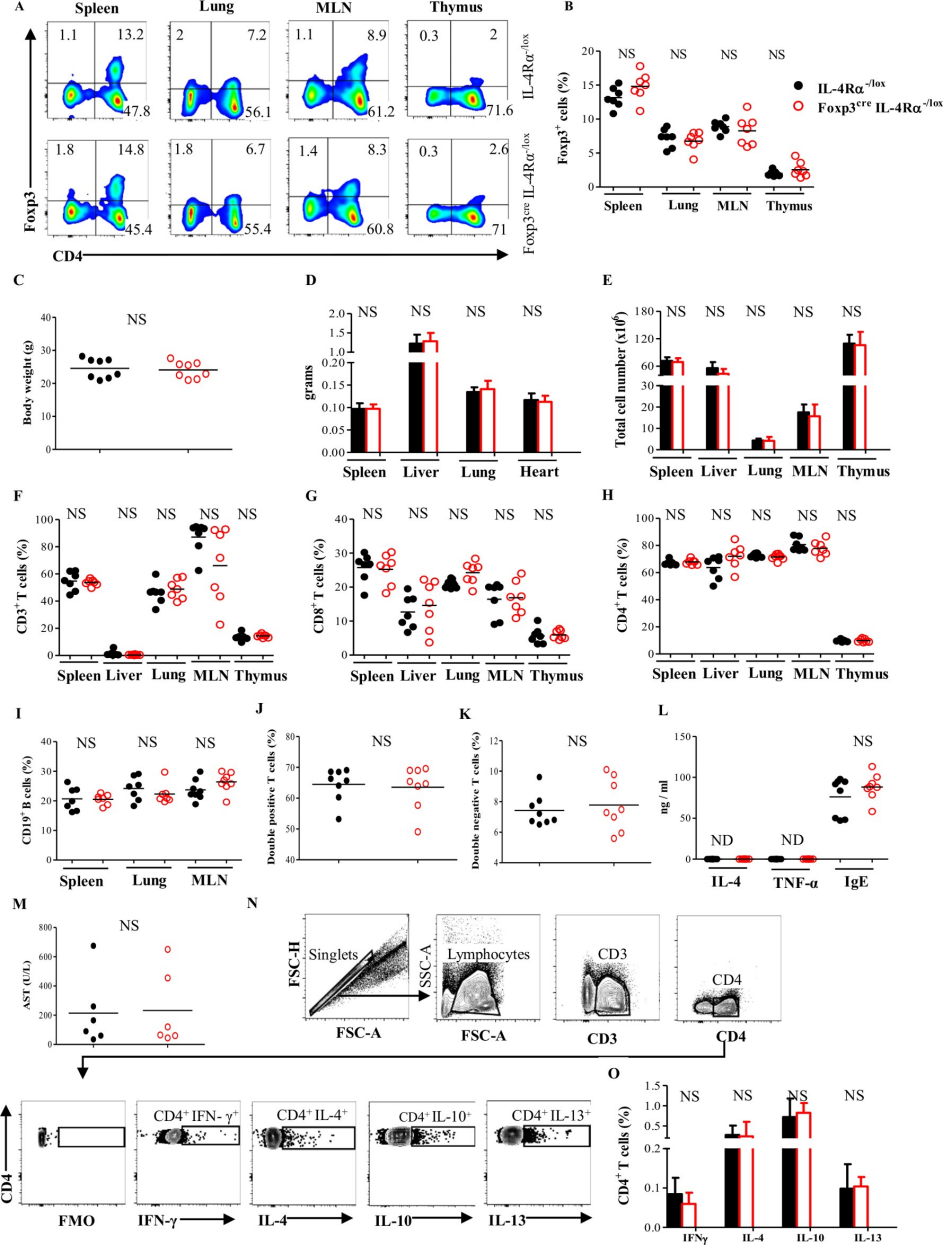
Deletion of IL-4Rα on Foxp3^+^ Treg cells does not break immune tolerance under a steady state. (A) Representative flow cytometry analysis of CD4^+^ Foxp3^+^ cells among CD3^+^ cells from spleen, lung, MLN, and thymus of naïve IL-4Rα^−/lox^ and Foxp3^cre^ IL-4Rα^−/lox^ mice. (B) Frequency of CD4^+^ Foxp3^+^ T cells from (A). (C) Body weight of naïve IL-4Rα^−/lox^ and Foxp3^cre^ IL-4Rα^−/lox^ mice. (D) Organ weights of naïve mice. (E) Total cell number of spleen, liver, lung, MLN, and thymus of naïve mice. (F) Frequency of CD3^+^, (G) CD3^+^ CD8^+^, and (H) CD3^+^ CD4^+^ T cells from organs of mice as in (E). (I) Frequency of CD19^+^ B cells in spleen, lung, and MLN of naïve mice. (J) Frequency of double-positive and (K) double-negative T cells in the thymus of naïve mice. (L) Serum analysis of naïve mice. (M) Analysis of liver function in naïve mice. (N) Gating strategy for identifying cytokine-producing CD4^+^ T cells. (O) Frequency of IFN-γ, IL-4, IL-10, and IL-13-expressing CD4^+^ T cells. MLN cells from naïve mice were restimulated with PMA/Ionomycin in the presence of monensin, after which CD4^+^ T cells stained intracellularly for indicated cytokines. Results are representative of two independent experiments with 7–9 mice/group. Data are expressed as mean ± S.E.M. NS, *P* > 0.05; * *P* < 0.05, ** *P* < 0.001, *** *P* < 0.0001 by two-tailed unpaired Student *t* test. Underlying data can be found in S1 Data. AST, aspartate aminotransferase; CD3, cluster of differentiation 3; CD4, cluster of differentiation 4; CD8, cluster of differentiation 8; CD19, cluster of differentiation 19; FMO, fluorescence minus one; Foxp3, forkhead box P3; FSC, forward scatter; IFN-γ, interferon gamma; IgE, immunoglobulin E; IL-4, interleukin-4; IL-10, interleukin-10; IL-13, interleukin-13; IL-4Rα, interleukin-4 receptor alpha; MLN, mesenteric lymph node; ND, not detectable; NS, not significant; PMA, phorbol myristate acetate; SSC-A, side scatter A; TNF-α, tumor necrosis factor alpha; Treg, regulatory T.

Equipped with the aforementioned murine model, we first interrogated the need for Foxp3^+^ Treg cells to express IL-4Rα expression under a steady state in vivo. Naïve Foxp3^cre^ IL-4Rα^−/lox^ mice and littermate control, IL-4Rα^−/lox^ mice, were examined for alteration of Foxp3^+^ Treg cell compartments, overall tissue pathologies, and tissue cellularities. Foxp3^cre^ IL-4Rα^−/lox^ mice had similar tissue frequencies of CD4^+^ Foxp3^+^ Treg cells among cluster of differentiation 3 (CD3)^+^ T cells when compared to their littermate control (Figs 3A and 3B and S3A). No aberrant changes in body weight (Figs 3C and S3B), vital organs’ weight (Figs 3D and S3C), or organs’ cellularities (Figs 3E and S3D) were noted. Foxp3^cre^ IL-4Rα^−/lox^ mice also displayed normal lymphocyte compartments in their primary lymphoid organ (thymus), secondary lymphoid organs (spleen and MLN), and peripheral tissues (lung and liver) (Fig 3F–3K and S3E–2I). Together, our findings suggest that no major physical alterations are consequent to the impairment of IL-4Rα-mediated signaling within the Foxp3^+^ T-cell compartment in our Foxp3^cre^ IL-4Rα^−/lox^ mouse model under a steady state.

Mice with a Treg cell–specific deletion of GATA3, a transcription factor closely associated with IL-4Rα-mediated signaling [24], have been shown to develop a spontaneous inflammatory disorder with an increased release of inflammatory soluble mediators [25]. To address whether the deletion of IL-4Rα within Foxp3^+^ Treg cells would also instruct a spontaneous and perhaps more subtle inflammatory response in Foxp3^cre^ IL-4Rα^−/lox^ mice, serum levels of soluble inflammatory mediators and liver enzymes and cytokine production by CD4^+^ T cells were determined. Serum levels of IL-4, tumor necrosis factor alpha (TNF-α), immunoglobulin E (IgE; Figs 3L and S3J), and aspartate aminotransferase (AST) (Figs 3M and S3K) in Foxp3^cre^ IL-4Rα^−/lox^ mice were similar to those of littermate controls. All CD4^+^ T cell–derived cytokines tested were also similar between Foxp3^cre^ IL-4Rα^−/lox^ mice and littermate controls (Figs 3N and 3O and S3L). Collectively, these results show that in vivo under a steady state, impairment of IL-4Rα-mediated signaling within the Foxp3^+^ T-cell compartment neither alters Foxp3^+^ Treg cell compartments nor results in spontaneous inflammatory disorder in Foxp3^cre^ IL-4Rα^−/lox^ mice.

To further address the molecular basis of Foxp3^+^ Treg cells’ need to express IL-4Rα signaling, we assessed the in vitro Foxp3 Treg conversion and expansion capacity of CD4^+^ CD25^−^/CD4^+^ CD25^+^ cells from Foxp3^cre^ IL-4Rα^−/lox^ mice, respectively, in comparison with CD4^+^ CD25^−^/CD4^+^ CD25^+^ cells from IL-4Rα^−/lox^ littermate controls. CD4^+^ CD25^−^ T cells were sorted with 99.8% purity (S4A Fig) from naïve Foxp3^cre^ IL-4Rα^−/lox^ mice and their IL-4Rα^−/lox^ littermate controls. Sorted cells were cultured in the presence of transforming growth factor beta 1 (TGF-β1) in combination with T-cell receptor stimulation using anti-CD3 and anti-CD28 for 72 hr (S4B and S4C Fig), which are known to induce conversion from CD25-negative to CD25-positive CD4^+^ T cells [26]. In our cultures, CD4^+^ CD25^−^ T cells converted to CD4^+^ CD25^+^ Foxp3^+^ T cells (induced Treg [iTreg] cells), with the highest rate of conversion recorded from CD4^+^ CD25^−^ T-cell cultures derived from Foxp3^cre^ IL-4Rα^−/lox^ mice (S4B and S4C Fig). This suggests that IL-4Rα signaling on CD4^+^ CD25^−^ T cells might interfere with their ability to convert but is clearly not needed to promote their differentiation into Foxp3^+^ Treg cell in vitro.

Next, we measured the effect of IL-4 stimulation on Treg cells. To do so, CD4^+^ CD25^+^ T cells, sorted from naïve Foxp3^cre^ IL-4Rα^−/lox^ mice and their littermate control (S4A Fig), were cultured in the presence or absence of rIL-4 for 18 or 36 hr. Whereas IL-4 treatment enhanced the survival of CD4^+^ CD25^+^ T cells from control mice, the survival of CD4^+^ CD25^+^ T cells from Foxp3^cre^ IL-4Rα^−/lox^ mice remained unaltered following treatment with rIL-4 (S4D Fig). These results suggest that IL-4/IL-4Rα signaling promotes CD25^+^ Treg cell survival in vitro and that this process is abrogated in CD25^+^ Treg cells from Foxp3^cre^ IL-4Rα^−/lox^ mice. Furthermore, CD4^+^ CD25^+^ Treg cells derived from IL-4Rα^−/lox^ mice considerably expanded Foxp3^+^ Treg cells’ frequency (S4E Fig) and expression level (S4F and S4G Fig) in response to in vitro stimulation with rIL-4, whereas CD4^+^ CD25^+^ Treg cells from Foxp3^cre^ IL-4Rα^−/lox^ mice failed to do so. Taken together, these data indicate that even though IL-4Rα-mediated signaling negatively influences the ability of naive CD4^+^ CD25^−^ Foxp3^−^ T cells to convert into Foxp3^+^ Treg cells, this receptor, later on, promotes the survival and enhances the Foxp3 expression of CD4^+^ CD25^+^ Treg cells in vitro.

### Intact IL-4Rα-mediated signaling on Foxp3^+^ Treg cell is required for the regulation of exacerbated immune responses

**Fig 4:**
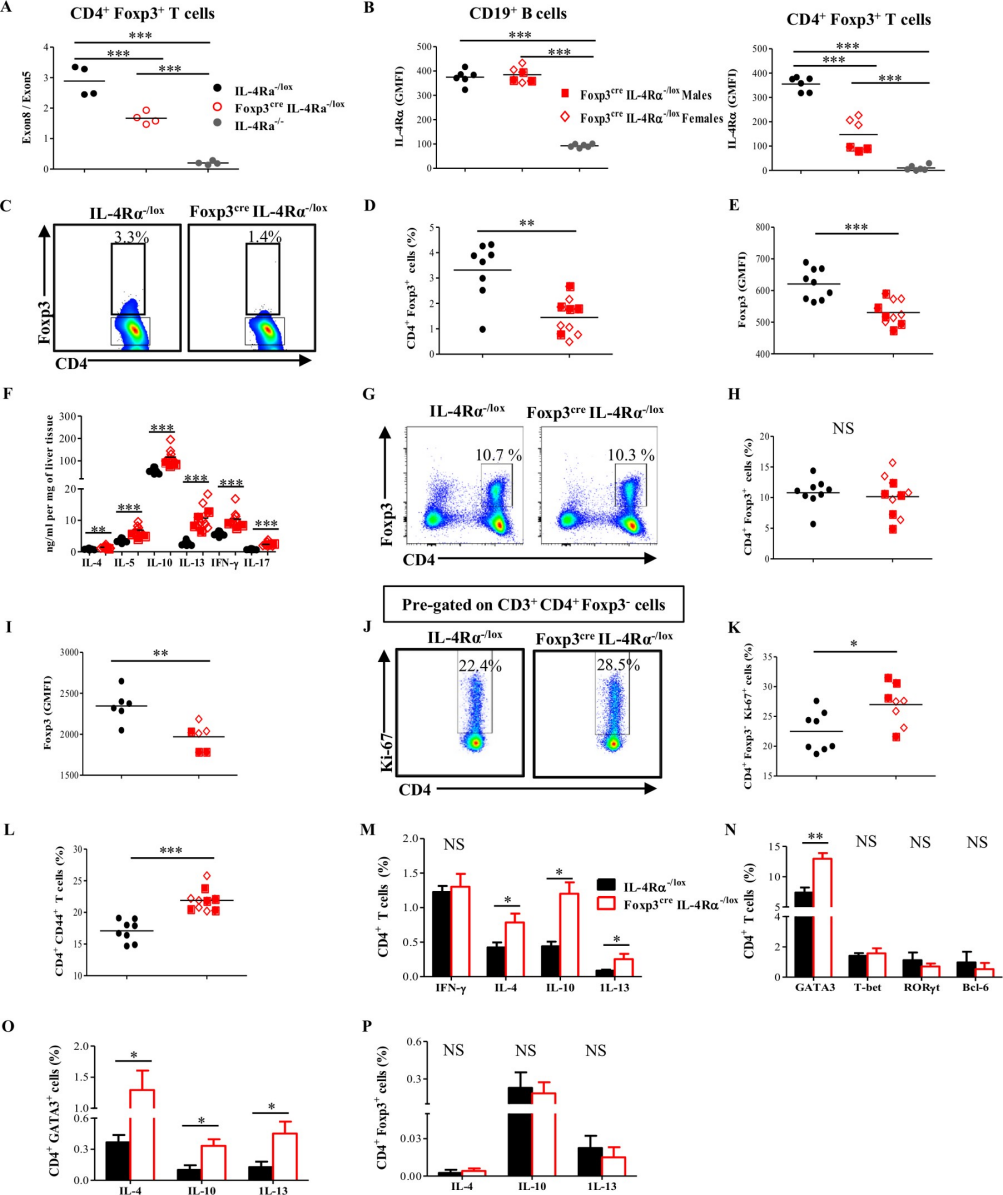
IL-4Rα-mediated signaling on Foxp3^+^ Treg cell is required for the accumulation of Treg cells and the control of effector T-cells. IL-4Rα^−/lox^, Foxp3^cre^ IL-4Rα^−/lox^, and IL-4Rα^−/−^ mice were infected with *Sm* cercariae and euthanized 8 wk post infection. (A) Efficiency of *il4ra* deletion by qPCR. CD4^+^ Foxp3^+^ T cells were sorted 8 wk post infection from pooled spleen and MLN cells, genomic DNA was extracted, and the ratio of exon8:exon5 was calculated. (B) Flow cytometry analysis of IL-4Rα expression by CD19^+^ B cell and CD4^+^ Foxp3^+^ T cell in pooled spleen and MLN cells 8 wk post infection. (C) Representative flow cytometry of CD4^+^ Foxp3^+^ T cells from the liver of mice infected with *Sm* for 8 wk. (D) Frequency of CD4^+^ Foxp3^+^ T cells (left) and (E) Foxp3 GMFI in CD4^+^ Foxp3^+^ T cells from (C). (F) Liver cytokine production 8 wk post infection. Livers from infected mice were homogenized, and the levels of the indicated cytokines were detected by ELISA and normalized to mg of liver tissue. (G) Representative flow cytometry of CD4^+^ Foxp3^+^ T cells from the MLN of mice infected with *Sm* for 8 wk. (H) Frequency of CD4^+^ Foxp3^+^ T cells (left) and (I) Foxp3 GMFI in CD4^+^ Foxp3^+^ T cells from (G). (J) Representative flow cytometry of CD4^+^ Ki-67^+^ cells within CD4^+^ Foxp3^−^ T-cell population in MLN 8 wk post infection. (K) Frequency of CD4^+^ Ki-67^+^ cells from (J). (L) Frequency of CD3^+^ CD4^+^ CD44^+^ effector T cells in MLN 8 wk post infection. (M) Frequency of cytokine-producing CD3^+^ CD4^+^ T cells, from MLN, after stimulation with PMA/Ionomycin in the presence of monensin. (N) Frequency of indicated transcription factor–expressing CD4^+^ T cells in the MLN 8 wk post infection. (O) Frequency of cytokine-producing CD4^+^ GATA3^+^ T cells, from MLN, after stimulation with PMA/Ionomycin in the presence of monensin. (P) Frequency of cytokine-producing CD4^+^ Foxp3^+^ T cells, from MLN, after stimulation with PMA/Ionomycin in the presence of monensin. Results are representative of two independent experiments with 6–10 mice/group. Data are expressed as mean ± S.E.M. NS, *P* > 0.05; * *P* < 0.05, ** *P* < 0.001, *** *P* < 0.0001 by two-tailed unpaired Student *t* test. Underlying data can be found in S1 Data. Bcl-6, B cell lymphoma 6; CD3, cluster of differentiation 3; CD4, cluster of differentiation 4; CD19, cluster of differentiation 19; CD44, cluster of differentiation 44; Foxp3, forkhead box P3; GATA3, GATA binding protein 3; GMFI, geometric mean fluorescence intensity; IFN-γ, interferon gamma; IL-4, interleukin-4; IL-4Rα, interleukin-4 receptor alpha; IL-5, interleukin-5; IL-10, interleukin-10; IL-13, interleukin-13; IL-17, interleukin-17; MLN, mesenteric lymph node; NS, not significant; PMA, phorbol myristate acetate; qPCR, quantitative real-time PCR; RORγt, RAR-related orphan receptor gamma; *Sm*, *S*. *mansoni*; T-bet; T-box transcription factor; Treg, regulatory T.

The strategy of probing subtle immune impairments, nonapparent during a steady state, under more inflammatory settings has previously proven to be efficient in unveiling hidden immune defects [5,27]. Moreover, the aforementioned enhancement of survival and Foxp3 expression of CD4^+^ CD25^+^ Treg cells upon induction of IL-4/IL-4Rα-mediated signaling in vitro and the striking higher expression of IL-4Rα by Foxp3^+^ Treg cells observed during infection warranted us to further address the possible higher need for this receptor by Foxp3^+^ Treg cells, in vivo, during inflammatory disease conditions. To do so, IL-4Rα^−/lox^, Foxp3^cre^ IL-4Rα^−/lox^, and IL-4Rα^−/−^ mice were infected with *Sm* cercariae and euthanized 8 wk thereafter. Deletion of *il-4rα* within the Foxp3^+^ Treg cell population was confirmed at the genomic level by qPCR (Fig 4A) and at the protein level by flow cytometry (Figs 4B, S5A and S5B), at which specific deletion of IL-4Rα on Foxp3^+^ Treg cells (more efficient in males, when compared to females), but not on CD19^+^ B cells, was observed. Flow cytometry analysis of Foxp3^+^ Treg cells within the *Sm*-diseased livers showed significant reduction of Foxp3^+^ Treg cell infiltration into the liver of *Sm*-infected Foxp3^cre^ IL-4Rα^−/lox^ when compared to their littermate controls (Figs 4C, 4D and S5C). Furthermore, we noted that Foxp3^+^ Treg cells in the liver of *Sm-*infected Foxp3^cre^ IL-4Rα^−/lox^ mice had a significant reduction in the expression levels of Foxp3 (Figs 4E and S5D). This suggests that IL-4Rα signaling seems to be necessary for the Foxp3^+^ Treg cell in order to maintain or up-regulate its expression of the suppressive marker Foxp3 in vivo during inflammation. Deletion of IL-4Rα signaling on Foxp3^cre^ IL-4Rα^−/lox^ mice resulted in elevated cytokine production, including Type 1 (IFN-γ), Type 17 (interleukin-17 [IL-17]), and Type 2 (IL-4, interleukin-5 [IL-5], interleukin-10 [IL-10], interleukin-13 [IL-13]) cytokines in the liver of both male and female *Sm-*infected Foxp3^cre^ IL-4Rα^−/lox^ mice (Figs 4F and S5E). These suggest that Foxp3^+^ Treg cells do require IL-4/IL-4Rα-mediated signaling in vivo to suppress overshooting inflammation.

The analysis within the MLNs supported this conclusion. Even though the frequency of Foxp3^+^ Treg cells (Figs 4G, 4H and S5G) within the MLNs in *Sm-*infected Foxp3^cre^ IL-4Rα^−/lox^ mice were similar to littermate controls at 8 wk post infection, Foxp3 surface expression level on a per-cell basis was, however, dramatically reduced in *Sm*-infected Foxp3^cre^ IL-4Rα^−/lox^ mice compared to infected littermate controls (Figs 4I and S5H). Reduced suppressive capacity potential was combined with increased frequency of proliferating (CD4^+^ Foxp3^−^ ki67^+^; Figs 4J, 4K and S5I) and effector (CD4^+^ cluster of differentiation 44 [CD44]^+^; Figs 4L and S5J) T cells within the MLN of *Sm*-infected Foxp3^cre^ IL-4Rα^−/lox^ mice, accompanied by significant increase in IL-4, IL-10, and IL-13 production by CD4^+^ T cells (Figs 4M and S5K). Together, these findings strongly suggest that the impairment of IL-4/IL-4Rα-mediated signaling within the Foxp3^+^ T-cell compartment diminishes Foxp3^+^ Treg cell recruitment to affected tissues during inflammation and impairs Treg cell ability to up-regulate suppressive factor, culminating into a heightened immune activation.

We next questioned the nature of the heightened immune response observed during inflammation in Foxp3^cre^ IL-4Rα^−/lox^ mice. To do so, MLN cells from *Sm*-infected Foxp3^cre^ IL-4Rα^−/lox^ mice and littermate controls were probed for CD4^+^ T cell–expressing GATA3 as a marker of Th2 responses [24], T-box transcription factor (T-bet) as a marker of T helper 1 (Th1) responses [28], RAR-related orphan receptor gamma (RORγt) as a marker of Th17 responses [29], and B cell lymphoma 6 (Bcl-6) as a marker of T follicular responses [30,31]. Consistent with the Th2 hegemony that is characteristic of *Sm* infection [32], MLN CD4^+^ T cells from *Sm*-infected Foxp3^cre^ IL-4Rα^−/lox^ mice overexpressed GATA3 (Figs 4N and S5L and S6A); however, all other T-cell polarization markers remained unaltered compared to littermate control (Figs 4N and S5L and S6A). Of importance, CD4^+^ GATA binding protein 3 (GATA3)^+^ cells from *Sm*-infected Foxp3^cre^ IL-4Rα^−/lox^ mice produced more IL-4, IL-10, and IL-13 compared to littermate controls (Figs 4O and S5M and S6B). Together, these suggest that CD4^+^ GATA3^+^ responses likely drive the heightened immune response and the elevated cytokine production observed in Foxp3^cre^ IL-4Rα^−/lox^ mice during inflammation. Nevertheless, with the recent description of ex-Foxp3 T cells [14,15,18], this observation could be confounded by the likelihood of cytokine production by newly formed ex-Foxp3^+^ T cells dually expressing GATA3 and Foxp3 and releasing cytokines following deletion of IL-4Rα. To address that, we traced back the source of the produced cytokines during *Sm* infection by co-staining of intracellular cytokines and Foxp3 in CD4^+^ T cells. We observed that CD4^+^ Foxp3^+^ T cells did not produce more cytokines in the MLN of *Sm*-infected Foxp3^cre^ IL-4Rα^−/lox^ mice (Figs 4P and S5N and S6B), ruling out a role for Foxp3^+^ T cells in the heightened cytokine production observed in *Sm*-infected Foxp3^cre^ IL-4Rα^−/lox^ mice. Collectively, we conclude that the heightened immune response observed—which is due to impairment of IL-4Rα-mediated signaling within the Foxp3^+^ T-cell compartment in *Sm*-infected Foxp3^cre^ IL-4Rα^−/lox^ mice—is driven at least in part by CD4^+^ GATA3^+^ Foxp3^−^ but not Foxp3^+^ T cells.

### Deletion of IL-4Rα within Foxp3^+^ Treg cell population impairs their ability to control tissue inflammation in diseases

**Fig 5:**
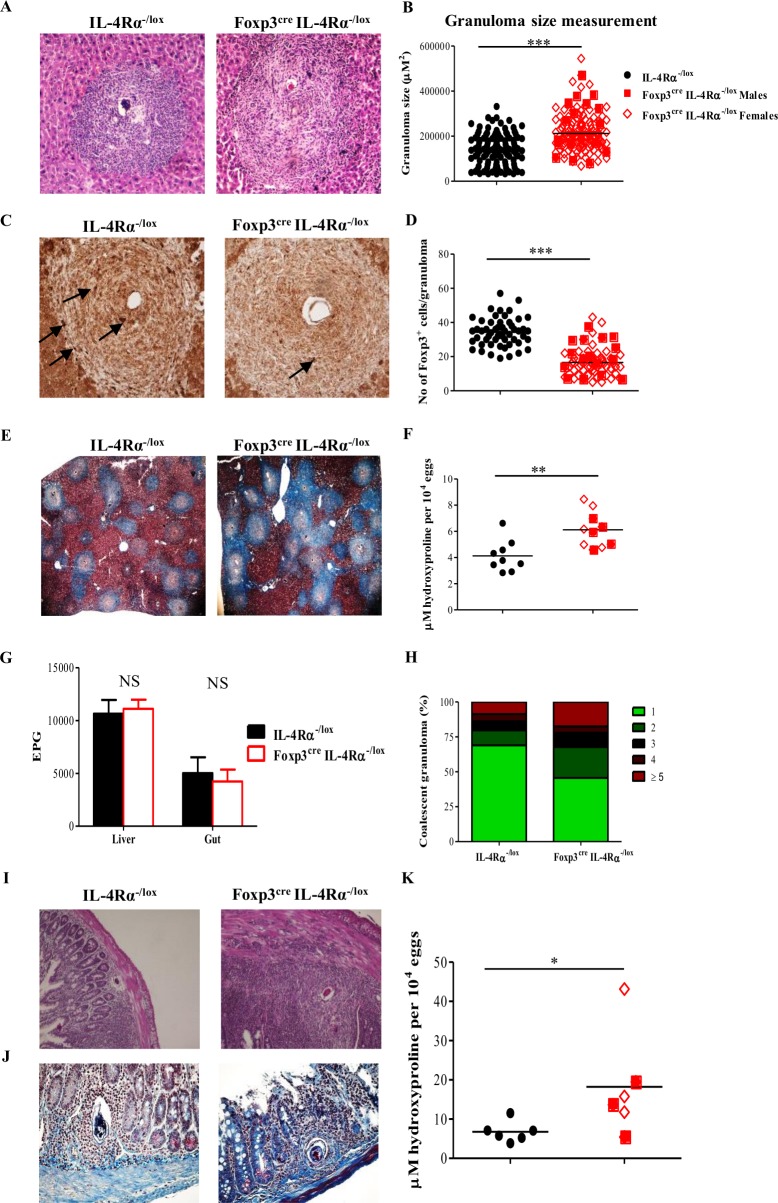
Deletion of IL-4Rα on Foxp3^+^ Treg cells impairs the control of fibrogranulomatous inflammation during schistosomiasis. (A) Representative HE staining of liver sections from mice infected with *Sm* for 8 wk (original magnification 100×). (B) Liver granuloma size. Granuloma size was determined from (A) by using a computerized morphometric analysis program (NIS elements by NIKON) by measuring 100 granulomas/group. (C) Representative Foxp3 Treg cells’ infiltration within liver granuloma 8 wk post *Sm* infection (original magnification 100×) of IL-4Rα^−/Lox^ and Foxp3^Cre^ IL-4Rα^−/Lox^ mice. Thin arrows point to Foxp3^+^ cells. (D) Quantification of the number of Foxp3^+^ cells per each granuloma from (C). (E) Representative CAB-stained liver sections from *Sm*-infected mice (original magnification 40×). (F) Liver hydroxyproline content measured by colorimetry 8 wk post infection. (G) Liver and gut egg burden 8 wk post infection. (H) Bar graphs showing the number of coalescent granulomas. (I) Representative HE staining of gut sections 8 wk post infection (original magnification 100×). (J) Representative CAB-stained gut sections 8 wk post infection (original magnification 200×). (K) Gut hydroxyproline content 8 wk post infection. Results are representative of two independent experiments with 6–10 mice/group. Data are expressed as mean ± S.E.M. NS, *P* > 0.05; * *P* < 0.05, ** *P* < 0.001, *** *P* < 0.0001 by two-tailed unpaired Student *t* test and one-way ANOVA with Bonferroni posttest analysis. Underlying data can be found in S1 Data. CAB, chromotrope aniline blue; EPG, egg per gram; Foxp3, forkhead box P3; HE, hematoxylin–eosin; IL-4Rα, interleukin-4 receptor alpha; NS, not significant; *Sm*, *S*. *mansoni*; Treg, regulatory T.

To further appraise the consequences of the heightened immune responses observed in diseased *Sm*-infected Foxp3^cre^ IL-4Rα^−/lox^ mice, egg-driven fibrogranulomatous inflammation, as well as Foxp3^+^ Treg cells’ infiltration in the liver of *Sm*-infected Foxp3^cre^ IL-4Rα^−/lox^ mice, was microscopically assessed in comparison with that of *Sm-*infected littermate controls. We noted that *Sm* infection resulted in enlarged egg-driven granulomas in the liver of Foxp3^cre^ IL-4Rα^−/lox^ mice when compared to granulomas in the liver of their littermate control (Fig 5A and 5B). In agreement with this observation, we found that the average number of Foxp3^+^ Treg cells within egg-driven granulomas in Foxp3^cre^ IL-4Rα^−/lox^ mice was significantly reduced when compared to the amount of Foxp3^+^ Treg cells recruited to the liver of littermate controls (Fig 5C and 5D). Furthermore, collagen levels, attesting the degree of tissue fibrosis, were considerably higher in the livers of *Sm*-infected Foxp3^cre^ IL-4Rα^−/lox^ mice when compared to the levels reported in *Sm*-infected littermate controls (Fig 5E). Indeed, colorimetric measurement of 4-hydroxyproline, a direct product of acid hydrolysis of collagen, confirmed our observed increased level of collagen in the liver of *Sm*-infected Foxp3^cre^ IL-4Rα^−/lox^ mice (Figs 4F and S5F). In fact, with similar egg burdens in the guts and livers of Foxp3^cre^ IL-4Rα^−/lox^ mice and their littermate control (Fig 5G), *Sm*-infected Foxp3^cre^ IL-4Rα^−/lox^ mice still displayed much larger coalescing granulomas (Fig 5H), as indicated by hematoxylin–eosin (HE) (Fig 5A) and chromotrope aniline blue (CAB) staining (Fig 5E). Similar to the liver, gut tissues of *Sm*-infected Foxp3^cre^ IL-4Rα^−/lox^ mice revealed elevated levels of cellular recruitment around trapped parasite eggs (Fig 5I), which was associated with a more pronounced deposition of collagen (Fig 5J), as confirmed by gut hydroxyproline content (Figs 5K and S5O), indicating a heightened fibrogranulomatous inflammation around *Sm*-trapped eggs in Foxp3^cre^ IL-4Rα^−/lox^ mice when compared to littermate controls.

Since Cre expression can generate a phenotype of its own [33–39], we sought to address whether the uncontrolled immune responses and the exaggerated fibrogranulomatous inflammation noted in the *Sm*-infected Foxp3^cre^ IL-4Rα^−/lox^ mice were due to the specific deletion of IL-4Rα on the Foxp3^+^ Treg cells or were an artefact effect from the Cre transgene alone. To test that, IL-4Rα^+/+^ and Foxp3^cre^ IL-4Rα^+/+^ mice were infected with 100 *Sm* cercariae, and then, Foxp3^+^ Treg cell compartments and fibrogranulomatous inflammation in liver and gut were investigated 8 wk post infection. We noted that IL-4Rα expression on either CD19^+^ B cells or Foxp3^+^ Treg cell compartment (S7A Fig) in *Sm*-infected Foxp3^cre^ IL-4Rα^+/+^ mice was similar to littermate controls. Furthermore, in liver, the frequency of Foxp3^+^ Treg cell population (S7B and S7C Fig) and level of Foxp3 (S7D and S7E Fig) as well as GATA3 (S7F and S7G Fig) expression within the Foxp3^+^ Treg cell compartment in *Sm-*infected Foxp3^cre^ IL-4Rα^+/+^ mice were similar or slightly affected when compared to their infected littermate controls. The similar frequency of Foxp3^+^ Treg cell population (S7H and S7I Fig), as well as the comparable level of Foxp3 expression (S7J and S7K Fig), was also noted in the MLN of *Sm*-diseased mice. In fact, Cre transgene expression in Foxp3^cre^ IL-4Rα^+/+^ mice led to neither an expansion of CD4^+^ GATA3^+^ T-cell population (S7L Fig) nor uncontrolled Type 1 (IFN-γ), Type 17 (IL-17), or Type 2 (IL-4, IL-5, IL-10, IL-13) cytokine production (S7M Fig). In agreement with these observations, we found that Cre expression did not affect either liver granuloma size (S8A and S8B Fig) or hepatic fibrosis, as indicated by CAB staining (S8C Fig) and colorimetric measurement of 4-hydroxyproline (S8D Fig). Similar to the liver, gut tissues of *Sm*-infected Foxp3^cre^ IL-4Rα^+/+^ mice had a comparable granuloma size (S8E Fig), similar collagen deposition (S8F Fig), and a similar level of 4-hydroxyproline content (S8G Fig) when compared to their infected littermate controls, indicating that Cre transgene, in our mouse model, does not have any impact on either the immune responses or the tissue inflammation.

Taken together, these observations indicate that the impairment of IL-4Rα-mediated signaling, but not the Cre transgene expression, specifically within the Foxp3^+^ Treg cell compartment during experimental schistosomiasis drives a poor accumulation of Foxp3^+^ T cells in the inflamed tissues and consequently results in elevated host fibrogranulomatous responses around the trapped parasite eggs.

We next aimed to test whether this would also hold true in another inflammatory helminth disease model. We found that following subcutaneous infection of Foxp3^cre^ IL-4Rα^−/lox^ and IL-4Rα^−/lox^ littermate control mice with *Nb*, the airways of *Nb*-infected Foxp3^cre^ IL-4Rα^−/lox^ mice showed much heavier mucus production 9 d post infection (S9A and S9B Fig). Moreover, we noted that Foxp3^+^ Treg cells’ recruitment to the inner layer of the alveoli of the lungs of *Nb-*infected Foxp3^cre^ IL-4Rα^−/lox^ mice (S9C and S9D Fig) was diminished, indicating that in this model as well the impairment of IL-4Rα-mediated signaling within the Foxp3^+^ T-cell compartment impairs their ability to accumulate at the inflamed tissue and to control local tissue inflammation. Collectively, these results clearly suggest, in two different and major inflammatory helminth models, that impairment of IL-4Rα-mediated signaling within the Foxp3^+^ T-cell compartment leads to uncontrolled immune responses and exacerbated tissue(s) inflammation, underscoring a hitherto-unappreciated role of IL-4Rα-mediated signaling on Foxp3^+^ Treg cells to control inflammatory responses in vivo.

### IL-4Rα-mediated signaling on Foxp3^+^ Treg cell is required for the generation of potent effector Foxp3^+^ Treg (eTreg) cell population

**Fig 6:**
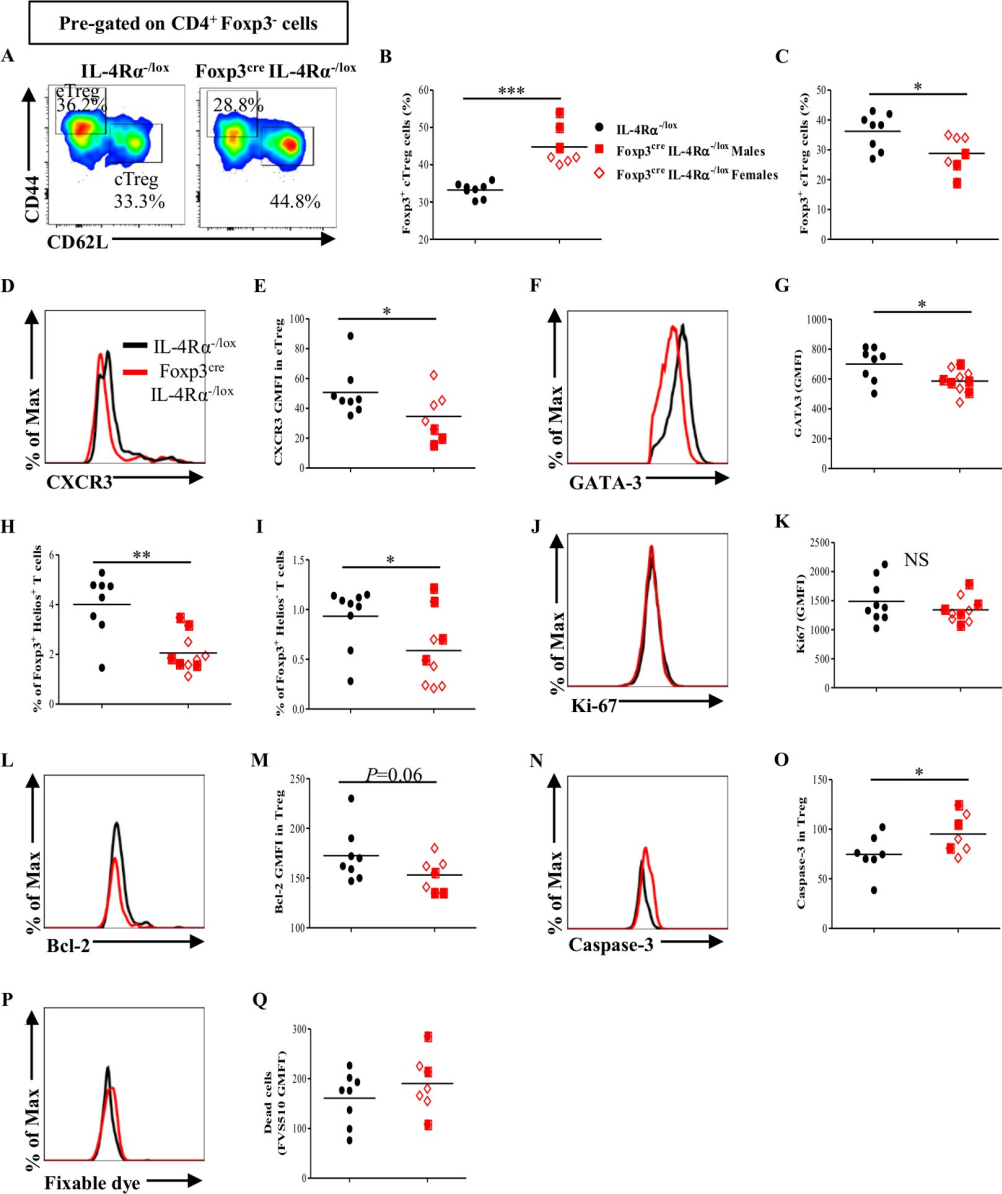
IL-4Rα-mediated signaling on Foxp3^+^ Treg cell is required for eTreg cell expansion, recruitment, and sustainability during schistosomiasis. (A) Representative flow cytometry of cTreg cells and eTreg cells from the hLN of mice infected with *Sm* for 8 wk. (B) Frequency of central CD4^+^ Foxp3^+^ Treg cells (left) and (C) effector CD4^+^ Foxp3^+^ Treg cells from (A). (D) Representative histogram of CXCR3 expression by effector CD4^+^ Foxp3^+^ T cells in the hLN 8 wk post infection with the mean values summarized in (E). (F) Representative histogram of GATA3 expression by CD4^+^ Foxp3^+^ T cells in the liver 8 wk post infection with the mean values summarized in (G). (H) Frequency of CD4^+^ Foxp3^+^ Helios^+^ Treg cells in the liver of *Sm*-infected mice. (I) Frequency of CD4^+^ Foxp3^+^ Helios^−^ Treg cells in the liver of *Sm*-infected mice. (J) Representative histogram of Ki-67 expression by CD4^+^ Foxp3^+^ T cells in the liver 8 wk post infection with the mean values summarized in (K). (L) Representative histogram of Bcl-2 expression by CD4^+^ Foxp3^+^ T cells in the liver 8 wk post infection with the mean values summarized in (M). (N) Representative histogram of caspase-3 expression by CD4^+^ Foxp3^+^ T cells in the liver 8 wk post infection with the mean values summarized in (O). (P) Representative histogram of fixable dye in CD4^+^ Foxp3^+^ T cells in the liver 8 wk post infection with the mean values summarized in (Q). Results pooled from two independent experiments with 3–4 mice/group. Data are expressed as mean ± S.E.M. NS, *P* > 0.05; * *P* < 0.05, ** *P* < 0.001, *** *P* < 0.0001 by two-tailed unpaired Student *t* test. Underlying data can be found in S1 Data. Bcl-2, B cell lymphoma 2; CD44, cluster of differentiation 44; CD62L, L-selectin; cTreg, central regulatory T; CXCR3, C-X-C motif chemokine receptor 3; eTreg, effector regulatory T; Foxp3, forkhead box P3; GATA3, GATA binding protein 3; GMFI, geometric mean fluorescence intensity; hLN, hepatic lymph node; IL-4Rα, interleukin-4 receptor alpha; NS, not significant; *Sm*, *S*. *mansoni*; Treg, regulatory T.

Next, we investigated the mechanisms underpinning the poor ability of Foxp3^+^ Treg cells to accumulate in the inflamed tissues in our diseased Foxp3^cre^ IL-4Rα^−/lox^ mice. First, we assessed the effect of IL-4Rα deletion specifically on Foxp3^+^ T-cell compartment for its ability to build up the eTreg cells needed to contain the immune responses at the site of inflammation [40]. To address that, central Foxp3^+^ Treg (cTreg) cell and eTreg cell populations in hepatic lymph nodes (hLNs) and MLNs of Foxp3^cre^ IL-4Rα^−/lox^ mice infected with *Sm* for 8 wk were assessed in comparison to those of *Sm-*infected littermate controls. In both lymph nodes, deletion of IL-4Rα specifically on Foxp3^+^ Treg cells resulted in the accumulation of cTreg cells (Figs 6A and 6B and S10A), which was associated with a significant reduction in the pool of eTreg cell population (Figs 6A and 6C and S10B), indicating that IL-4Rα-mediated signaling on Foxp3^+^ Treg cells could be playing a role in inducing the conversion of cTreg cell to eTreg cell. Furthermore, the ability of these eTreg cells to maintain or up-regulate the expression of C-X-C motif chemokine receptor 3 (CXCR3), a chemoattractant receptor required for the migration of eTreg cells to nonlymphoid tissues (in particular, liver [40,41]), was significantly impaired in both hLNs (Fig 6D and 6E) and MLNs (S10C and S10D Fig) of Foxp3^cre^ IL-4Rα^−/lox^ mice infected with *Sm* for 8 wk. In support, the expression level of GATA3, a transcription factor required for Treg cell accumulation at the site of inflammation [42], within the Foxp3^+^ Treg cell in the liver of Foxp3^cre^ IL-4Rα^−/lox^ mice infected with *Sm* for 8 wk was diminished when compared to littermate controls (Fig 6F and 6G). Together, these results suggest that IL-4Rα-mediated signaling on Foxp3^+^ Treg cell is required for the maintenance or up-regulation of the markers required for migration and accumulation of Foxp3^+^ Treg cell in the inflamed tissue.

Then, we traced back whether the reduction in the Foxp3^+^ Treg cell population in the liver of Foxp3^cre^ IL-4Rα^−/lox^ mice infected with *Sm* for 8 wk was due to a global defect in Foxp3^+^ Treg cells or in the Treg cell de novo conversion in vivo, since eTreg cell can be thymic-derived and/or peripherally induced. To address that, we analyzed the thymic-derived Foxp3^+^ Treg cells (Foxp3^+^ Helios^+^ Treg cells) and the peripherally iTreg cells (Foxp3^+^ Helios^−^ Treg cells) in the liver of Foxp3^cre^ IL-4Rα^−/lox^ mice infected with *Sm* for 8 wk and their littermate controls. We noted that in the liver of Foxp3^cre^ IL-4Rα^−/lox^ mice infected with *Sm* for 8 wk, both Foxp3^+^ Helios^+^ and Foxp3^+^ Helios^−^ Treg cells were significantly reduced (Fig 6H and 6I), suggesting a global defect in the Foxp3^+^ Treg cell population.

Finally, inasmuch as the reduction of Foxp3^+^ Treg cell population was independent of its proliferation as Foxp3^+^ Treg cells in the liver of Foxp3^cre^ IL-4Rα^−/lox^ mice infected with *Sm* for 8 wk and their littermate control had a comparable expression level of Ki-67 (Fig 6J and 6K), we therefore asked whether the reduction of Foxp3^+^ Treg cell population in the liver could be possibly driven, in addition to their poor migration and accumulation, by the lessened ability of these Foxp3^+^ Treg cells to survive in the absence of IL-4Rα-mediated signaling. Indeed, we noted a diminished expression level of the pro-survival factor B cell lymphoma 2 (Bcl-2; Fig 6L and 6M) within the Foxp3^+^ Treg cell population in the liver of the Foxp3^cre^ IL-4Rα^−/lox^ mice infected with *Sm* for 8 wk when compared to their littermate control. In support, those Foxp3^+^ Treg cell population had a higher expression level of the apoptotic marker caspase-3 (Fig 6N and 6O) as well as the fixable viability stain (Fig 6P and 6Q), indicating that the Foxp3^+^ Treg cell population in the liver has a higher propensity to undergo apoptosis in the absence of IL-4Rα-mediated signaling, which recapitulates our in vitro data mentioned earlier. Collectively, our results suggest that IL-4Rα-mediated signaling is required for the conversion of cTreg cells into eTreg cells. These eTreg cells further need the IL-4Rα-mediated signaling to maintain or up-regulate markers required for their migration, accumulation, and survival at the site of inflammation.

## Discussion

**Fig 7:**
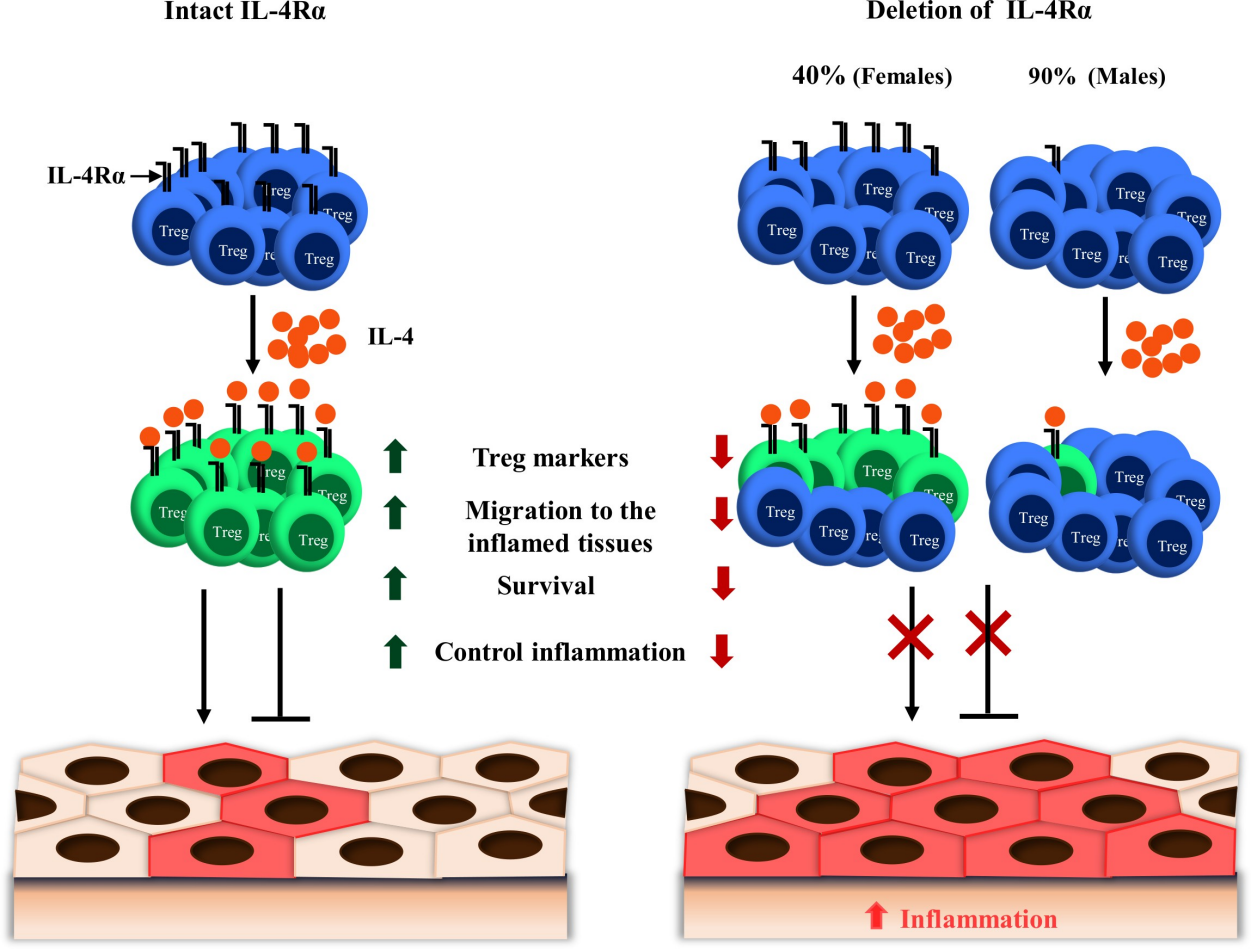
IL-4Rα is required by Treg cells to control inflammation in disease. Schematic diagram showing that either partial or complete deletion of IL-4Rα from Foxp3^+^ Treg cells leads to aggravated pathology and uncontrolled inflammation, but rather, Foxp3^+^ Treg cells do require an intact IL-4Rα signaling to control inflammation in diseases. Foxp3, forkhead box P3; IL-4, interleukin-4; IL-4Rα, interleukin-4 receptor alpha; Treg, regulatory T.

Studies on Foxp3^+^ Treg cell function have uncovered a critical regulatory role for the cytokines present in the milieu (reviewed in [9]). IL-4, the canonical cytokine of Type 2 immune responses that signals via IL-4Rα, signaling on Treg cells has been shown to promote Treg cell reprogramming toward Th17 [15] or Th2 [14,18] cells. As a consequence, enhanced signaling via IL-4Rα in Treg cells has resulted in impaired suppressive function and heightened inflammation, establishing the concept of a negative regulation of Treg cell activity by IL-4Rα-mediated signaling. The present study, however, shows that IL-4Rα-mediated signaling is also required by Treg cells to control inflammation in diseases (Fig 7). First, in an experimental murine model of schistosomiasis, we demonstrated that Foxp3^+^ Treg cells up-regulated IL-4Rα expression. Further analyses on the need for the host to up-regulate this receptor during schistosomiasis were then conducted using a murine model of specific deletion of IL-4Rα within the Foxp3^+^ Treg cell population, termed Foxp3^cre^ IL-4Rα^−/lox^ mice. Specific removal of IL-4Rα in a part or almost totally on the Foxp3^+^ Treg cell population exacerbated tissue inflammation during experimental *Sm*- and *Nb*-mediated diseases. Aggravated immunopathology was associated with a decline in Treg cell accumulation in the diseased tissues and the reduced expression of suppressive markers, paralleled by an augmentation of immune effector responses as translated in the *Sm* model in Foxp3^cre^ IL-4Rα^−/lox^ mice. Collectively, these findings unprecedentedly, to our knowledge, uncovered a critical need for IL-4Rα-mediated signaling on Foxp3^+^ Treg cells to control tissue immunopathology in helminthiases. Such a need for Foxp3^+^ Treg cells for this signaling axis is yet to be examined in allergic Th2 inflammation and Th1- or Th17-dominated diseases.

Recently published reports have shown that augmentation of IL-4Rα-mediated signaling on Foxp3^+^ Treg cells leads to an impaired suppressive activity of those cells with subsequent reprogramming into ex-Foxp3 Th2 or Th17 cells [14,15,18]. Although these observations would suggest that IL-4Rα signaling might not favor Treg cell activity, our present analyses revealed that Foxp3^+^ Treg cells have a basal expression level of IL-4Rα under a steady state, pointing at a possible requirement for this receptor in Treg cell biology. Our assumption is in accordance with a previous attempt by other investigators that have reported the potency of IL-4 to prevent spontaneous apoptosis and reduction of Foxp3 expression in the cultures of isolated Treg cells [10]. In fact, IL-4 stimulation of isolated cultures of CD4^+^ CD25^+^ Treg cells in our study recapitulated such a need for IL-4Rα-mediated signaling to prevent cell death and to maintain or enhance Foxp3 expression, indicating a hitherto-unappreciated need for IL-4Rα-mediated signaling in Treg cell biology and activity. IL-4Rα expression by Foxp3^+^ Treg cells was further enhanced following *Sm* infection, suggesting a strong requirement for this receptor either to preserve Treg cell activity or foster their transdifferentiation into ex-Foxp3 Th2 cells. In fact, the reprogramming of Foxp3^+^ Treg cells into functional effector ex-Foxp3 Th2 cells has now been demonstrated during *Heligmosomoides polygyrus* infection [18], during which ex-Foxp3 Th2 cells generated in an IL-4Rα-dependent manner were shown to contribute to the overall antiparasitic Th2 response [18]. Hence, it is possible that IL-4Rα up-regulation on Foxp3^+^ Treg cells during acute experimental schistosomiasis observed in our study could occur as an attempt for the host to rapidly mount a protective Th2 immune response during acute schistosomiasis [32]. More experiments are clearly needed at this point to address the likelihood of such a contribution of ex-Foxp3 in the host protective Th2 response during experimental schistosomiasis.

IL-4Rα removal specifically from the Foxp3^+^ Treg cell compartment was achieved by making use of the Cre-lox system. A murine model, Foxp3^cre^ IL-4Rα^−/lox^, was obtained in which IL-4Rα was deleted specifically within the Foxp3^+^ Treg cell compartment in a dosage-dependent manner whereby IL-4Rα is partially deleted in females and quasi-completely deleted in males. The more efficient Cre-mediated IL-4Rα deletion achieved in male when compared to female Foxp3^cre^ IL-4Rα^−/lox^ mice strongly fits the phenomenon of random inactivation of the X chromosome that can take place in females but not males, as previously reported [43,44]. Our approach herein described could therefore set a precedent for the generation of transgenic mouse models with various levels of knockdown of a target gene.

We found, consistent with the literature [10], that isolated cultures of CD25^+^ Treg cells tightly depended on IL-4Rα for survival and maintenance of Foxp3 expression. However, partial or quasi-complete removal of IL-4Rα from the Foxp3^+^ Treg cell population did not affect the Treg cell compartment during steady state. A possible explanation could be that the remnant IL-4Rα on Foxp3^+^ Treg cells in our Foxp3^cre^ IL-4Rα^−/lox^ model is sufficient to preserve the Foxp3^+^ Treg cell compartment and thus tolerance during a steady state in which randomly occurring inflammatory responses are not common. This is consistent with previous studies that have attempted to define the physiological importance of factors expressed by Foxp3^+^ Treg cells. Specifically, in these studies, Foxp3-specific GATA3- or T-bet-deficient mice were clinically indistinguishable from their littermate controls and showed no defect on the function of Foxp3^+^ Treg cells under a steady state in young mice [5,27,42]. In older mice (6 mo upwards), however, the targeting of GATA3 within the Foxp3^+^ Treg cell population led to inflammatory disease [25]. It is therefore feasible that specific deletion of IL-4Rα from the Foxp3^+^ Treg cell compartment might also lead to apparent physiological defects only in old mice, but this is still to be addressed experimentally. Notably, inflammation in these models of Foxp3-specific GATA3 or T-bet deletion uncovered more robustly a defect in the Foxp3^+^ Treg cell compartment that translated into physiological impairments [27]. Similarly, infection of our Foxp3^cre^ IL-4Rα^−/lox^ mice resulted in elevated inflammatory responses in helminth-mediated disease models. This was defined by increased levels of Foxp3^−^ T-cell proliferation and activation as well as a clear augmentation of cytokine production, indicating a more potent effector response following either partial or quasi-complete deletion of IL-4Rα from the Foxp3^+^ Treg cell compartment during inflammation. It is also important to point out that we observed similarly heightened cytokine release and inflammation in diseased female Foxp3^cre^ IL-4Rα^−/lox^ mice, in which the less efficient deletion of IL-4Rα was observed when compared to *Sm*-infected male Foxp3^cre^ IL-4Rα^−/lox^ mice. The possible explanations could be that (i) disruption of the IL-4Rα signaling pathway on Foxp3^+^ Treg cells as low as 40% is enough to completely disequilibrate the inflammation–regulation balance during infection, and/or (ii) the functional characterization of males and females mice, by using STAT6 phosphorylation as a readout, demonstrated that the absolute final levels of STAT6 phosphorylation in cells from male and female Foxp3^cre^ IL-4Rα^−/lox^ mice were comparable (S1C Fig), indicating that male and female Foxp3^cre^ IL-4Rα^−/lox^ mice do have a similar defect at the functional level of the IL-4Rα signaling pathway on Foxp3^+^ Treg cell population. Such observations could provide an explanation for the similar phenotype in Foxp3^cre^ IL-4Rα^−/lox^ female and male mice during inflammation, when elevated IL-4 is produced.

As of yet, the general observation of heightened inflammatory responses in diseased Foxp3-specific IL-4Rα-impaired mice could be rooted under two opposing but not mutually exclusive explanations—i.e., (i) IL-4Rα as a critical receptor in conferring to Foxp3^+^ Treg cells the necessary potency to control inflammatory responses in disease and/or (ii) the need for this IL-4Rα receptor to foster Foxp3^+^ Treg cells transdifferentiation into effector T cells. The former explanation does find support in our observation of a significant reduction in Foxp3 expression levels within the Foxp3^+^ Treg cell population following removal of IL-4Rα from the Foxp3 compartment in *Sm*-infected Foxp3^cre^ IL-4Rα^−/lox^ mice. In fact, Foxp3 expression is the defining factor that endows Treg cell with a suppressive ability, and its continuous expression is required to maintain the transcriptional and functional programs of Treg cells [45–48]. Consequently, our observation of a reduced Foxp3 expression during inflammation following IL-4Rα removal on Foxp3^+^ Treg cells defines a need for this receptor in stabilizing and promoting Foxp3 expression by Foxp3^+^ Treg cells in vivo. Although the latter explanation of a negative regulation of Foxp3^+^ Treg cells by IL-4Rα signaling in favor of ex-Foxp3 transdifferentiation can be appropriately dismissed by using fate-reporter mice, our present study, as it stands, convincingly indicates that the T cells responsible for the increased cytokine production reported in Foxp3^cre^ IL-4Rα^−/lox^ diseased mice expressed not Foxp3 but rather GATA3, a profile inconsistent with recently reported ex-Foxp3 T cells during helminth infections [18]. This argued against Foxp3 Treg cells’ transdifferentiation into cytokine-producing effector T cells as mediating the heightened inflammation observed in diseased Foxp3^cre^ IL-4Rα^−/lox^ mice.

Partial or quasi-complete removal of IL-4Rα within the Foxp3^+^ Treg cell compartment of Foxp3^cre^ IL-4Rα^−/lox^ diseased mice led to an impaired accumulation of Treg cells to the site of inflammation. Mechanistically, various processes can mediate such an impaired accumulation of Foxp3^cre^ IL-4Rα^−/lox^ Treg cells observed in our infectious settings, including defects in (i) the extrathymic Treg cell conversion, (ii) the proliferation capacity, (iii) the generation of eTreg cells, (iv) the migration and the accumulation of eTreg cells at the site of inflammation, and/or (v) the survival of Treg cells. We found no obvious defect of CD25^−^ CD4^+^ cell precursors from Foxp3^cre^ IL-4Rα^−/lox^ mice in converting into Foxp3^+^ Treg cells in vitro, arguing against a defect in extrathymic Treg cell conversion. Ex vivo analyses of Foxp3^+^ Treg cells from diseased Foxp3^cre^ IL-4Rα^−/lox^ mice showed no signs of impaired proliferation, dismissing Foxp3^+^ Treg cell–defective proliferation as a possible cause of the poor accumulation reported. However, we found that cTreg cells in the lymph nodes of diseased Foxp3^cre^ IL-4Rα^−/lox^ mice had a poor ability to convert into eTreg cells, resulting in a significant reduction in the eTreg cell population required to populate the nonlymphoid tissues that further contain the inflammatory immune responses [40]. Furthermore, migration and accumulation of these eTreg cells were impaired, as indicated by the reduction in the expression level of CXCR3 and GATA3, respectively. Our results are in full accordance with previous reports on the need of Foxp3^+^ Treg cells to express GATA3 and CXCR3 to migrate and accumulate in the inflamed tissues and in the liver, respectively [9,41,42]. In addition to the reduction in the pool of eTreg cell population and their poor ability to migrate and accumulate at the site of inflammation, Foxp3^+^ Treg cells in the liver of diseased Foxp3^cre^ IL-4Rα^−/lox^ mice had a higher propensity to undergo apoptosis, recapitulating our in vitro data, which showed that CD25^+^ Treg cells’ survival and stability (as judged by Foxp3 expression) are compromised in the absence of IL-4.

Overall, our results support the idea that an intact, rather than a potentiated or diminished, IL-4Rα-mediated signaling is optimal to endow Foxp3^+^ Treg cells with their most efficient ability to control inflammation in disease as illustrated here in helminthiases. As per our current findings, such an optimal response is most likely achieved by preventing Foxp3^+^ Treg cell death and stabilizing the expression of canonical Treg cell–suppressive and associated markers such as Foxp3 and GATA3. In light of past reports that have defined a negative role for IL-4Rα-mediated signaling on Treg cell activity in Th2-dominated settings, our present finding of a negative role of IL-4Rα deficiency on Treg cells during inflammation further highlights the fact that the same cytokine can have both positive and negative effects on Treg cell activity and reinstates the notion that balance is key. Promoting or impairing IL-4Rα-mediated signaling yielded a similar result of Foxp3^+^ Treg cell impairment, therefore strongly arguing against the benefits of uninformedly modulating this cytokine receptor on Foxp3^+^ Treg cells to ameliorate tolerance, particularly in the current global context where helminthiasis are highly common. This is therefore particularly important in light of recent therapeutic advances for the control of highly pathogenic cases of asthma for which IL-4Rα targeting has recently been introduced and is attracting further interest for other inflammatory conditions [14,15].

Conclusively, in this study, a case is made for the preservation of intact levels of IL-4Rα signaling on Foxp3^+^ Treg cells to ensure optimal regulatory responses in vivo, and caution is therefore raised for the careful manipulation of this signaling axis in disease.

## Material and methods

### Ethics statement

All mice were maintained in specific-pathogen-free barrier conditions in individually ventilated cages at the University of Cape Town biosafety level 2 animal facility. Experimental mice were sex- and age-matched and used between 6 and 8 wk of age. All the experimental work was in strict accordance with the recommendations of the South African national guidelines and of the University of Cape Town practice for laboratory animal procedures, as in ethics protocols 014/003 and 016/027 approved by the Animal Research Ethics Committee of the Faculty of Health Science, University of Cape Town. All efforts were made to minimize animals’ suffering. Prior to percutaneous infection with *Sm* cercariae, animals were anesthetized by intraperitoneal injection of a cocktail of ketamine (100 mg/kg) and xylazine (10 mg/kg) and monitored for 5 min to confirm deep anesthesia. Anesthesia was confirmed by the absence of pedal reflex (toe pinch) and eyeblink reflex amid a regular respiratory rate. The anesthesia duration was of a maximum of 30 min. During the anesthesia phase, animals were exposed to an infrared lamp to help them maintain their core body temperature. This procedure was performed and duly cared for by trained and authorized researchers. Post infection, animals were monitored until regaining of consciousness, and moistened food was added to the cage bedding. Upon reaching the study’s experimental end point and/or the protocol-defined humane end point, animals were euthanized under this study by exposure to an excess of halothane (4% in air) for 5 minutes. Death was confirmed either by neck dislocation or exsanguination by cardiac puncture. Death was not a predetermined end point in any of the arms of this study.

### Mice

IL-4Rα^+/+^, IL-4Rα^−/−^, and IL-4Rα^−/lox^ mice on BALB/c background and C57BL/6 mice were previously described [22,23]. For Foxp3^cre^ IL-4Rα^−/lox^ mice, transgenic Foxp3^cre^ mice (a generous gift from Prof. James Wing, Osaka University) were intercrossed for two generations with IL-4Rα^−/−^ BALB/c mice [22]. These mice were further intercrossed with homozygous IL-4Rα^lox/lox^ BALB/c mice [23] to generate hemizygous Foxp3^cre^ IL-4Rα^−/lox^ mice BALB/c strain. Hemizygous littermates (IL-4Rα^−/lox^) were used as wild-type controls in all experiments. Mice were genotyped as described previously [22,23].

### Mice infection and challenge

#### *Sm* infection

Mice were percutaneously infected via the abdomen, using stainless steel rings, with 100 viable cercariae of a Puerto Rican strain of *Sm* obtained from infected *Biomphalaria glabrata* snails (NMRI strain, NR-21962, provided by Biomedical Research Institute, Rockville, MD, United States). Mice were euthanized 8 wk post infection, and eggs isolated from liver and ileum were counted as previously described [49].

#### *Nb* infection

Mice were injected subcutaneously with 500 *Nb* L3 larvae suspended in 0.9% NaCl using a 21-G needle (Braun, Melsungen, Germany). Mice were euthanized 9 d post infection, and tissue samples were collected for analyses.

### Cell isolation

Thymus and MLN single-cell suspensions were prepared by mechanical squeezing through a 40 μM cell strainer (Falcon, Corning, MA, US). Single-cell spleen suspensions were prepared by mechanical squeezing through a 70 μM cell strainer (Falcon). Liver lymphomyeloid cells were isolated following a modified version of the method of Gossen and colleagues and Hardy and colleagues [50,51]. Briefly, single-cell suspensions from the liver tissues were prepared by chopping them into 10 mm^3^ small pieces and incubating them in Iscove’s Modified Dulbecco’s Medium (IMDM) containing 220 U/mg Collagenase I (Gibco, Waltham, MA, US), 13 U/mg DNase I (Sigma, St. Louis, MO, US), and 5% inactivated fetal calf serum (iFCS; Gibco) (digestion buffer) for 30 min at 37°C under constant rotation. The resulting suspension was mechanically squeezed through a 100 μM sterile cell strainer (Falcon), followed by centrifugation at 1,200 rpm for 10 min at 4°C. Supernatant was discarded and the cells resuspended in 36% of isotonic Percoll (Sigma). This suspension was mixed thoroughly, and separation was performed at 500 *g*, without breaks, for 10 min at 4°C. For lungs, single-cell suspensions were prepared by chopping the lungs into 10 mm^3^ pieces, incubating them in digestion buffer for 30 min at 37°C, and mechanically squeezing them through a 70 μM cell strainer. Erythrocytes were lysed using RBC lysis buffer. Cells were washed; resuspended in IMDM containing 10% iFBS, 100 U/ml penicillin (Gibco), and 100 μg/ml streptomycin (Gibco) (IMDM culture medium); and checked for viability and cell number by trypan blue staining.

### Flow cytometry

Antibodies used for flow cytometry analysis were as follows: IL-4Rα (mIL4R-M1), CD3ε (500A2), CD4 (RM4-5), CD8α (53–6.7), CD25 (7D4), CD19 (1D3), Lineage, Foxp3 (FJK-16s), GATA3 (L50-823), T-bet (eBio4B10), Bcl-6 (K112-91), RoRγt (Q31-378), Helios (22F6), IRF4 (irf4.3e4), Ki67 (B56), Bcl-2, caspase-3 (C92-605), FVS dye, CXCR3 (CXCR3-173), CD44 (IM7), CD62L (MEL-14), IFN-γ (XMG1.2), IL-4 (11B11), IL-10 (JES5-16E), IL-13 (eBio13A), and p-STAT6 (J71-773.58.11) purchased from BD Biosciences (Franklin Lakes, NJ, US) and eBioscience (San Diego, CA, US). For staining of cell-surface markers, cells (1 × 10^6^) were labeled and washed in PBS containing 1% BSA (Roche, Switzerland) and 0.1% NAN_3_ (FACS buffer). For detection of intracellular cytokines, cells were seeded at a density of 2 × 10^6^ cells/well in complete IMDM culture medium and stimulated with 50 ng/ml phorbol myristate acetate (PMA), 250 ng/ml Ionomycin, and 200 μM monensin (all from Sigma) for 8–12 hr at 37°C in a humidified atmosphere containing 5% CO_2_. After the incubation period, cells were harvested, washed, fixed in 2% (w/v) paraformaldehyde, permeabilized with 0.5% saponin buffer, and then stained for cytokine production as previously described [23]. For intranuclear staining, BD Pharmingen Transcription Factor Buffer Set (BD Biosciences) was used as per manufacturer's instruction for detection of transcription factors. Acquisition was performed using BD LSRFortessa (BD Biosciences), and data were analyzed using FlowJo software (Treestar, Ashland, OR, US).

### Genotypic evaluation of efficiency and specificity of IL-4Rα deletion

Genomic DNA was isolated from CD3^+^ CD4^+^ Foxp3^−^, CD3^+^ CD4^+^ Foxp3^+^ T cells and CD19^+^ B cells sorted using BD FACSAria III cell sorter (BD Biosciences). Cell purity determined by flow cytometer amounted to at least 99%, and NanoDrop (ND-1000 Spectrophotometer, Thermo Fischer Scientific) was used for measuring DNA concentration and purity. Efficiency of *il-4rα* gene deletion was quantified by qPCR on LightCycler 480 Instrument II (Roche) using the following primers; exon 5: forward 5′ AACCTGGGAAGTTGTG 3′ and reverse 5′ CACAGTTCCATCTGGTAT 3′, exon 8: forward 5′ GTACAGCGCACATTGTTTTT 3′ and reverse 5′ CTCGGCACTGACCCATCT 3′. PCR conditions were 94°C for 2 min, 94°C for 20 s, 45°C for 30 s, and 72°C for 20 s for 55 cycles.

### Cell culture

Pooled cells from **s**pleen and MLN from naïve Foxp3^cre^ IL-4Rα^−/lox^ mice and their littermate control were cultured at 5 × 10^6^ cells/ml in RPMI medium (Lonza, Walkersville, MD, US) supplemented with 10% iFBS and penicillin and streptomycin (100 U/ml and 100 μg/ml) (RPMI culture medium) for 40 hr at 37°C in a humidified atmosphere containing 5% CO_2_, in medium supplemented with 0 or 1 ng/ml rIL-4 (BD Biosciences). For measuring the level of STAT6 phosphorylation, **s**pleen and MLN cells from naïve Foxp3^cre^ IL-4Rα^−/lox^ mice, their littermate control, and global knock-out mice were cultured at 1 × 10^6^ cells/ml RPMI culture medium for 1 hr at 37°C in a humidified atmosphere containing 5% CO_2_, in medium supplemented with 0 or 10 ng/ml rIL-4. After the incubation period, cells were harvested, washed in FACS buffer, and then stained for IL-4Rα and/or p-STAT6. IL-4Rα expression and level of STAT phosphorylation were detected by flow cytometry. In other settings, sorted CD4^+^ CD25^+^ T cells from naïve Foxp3^cre^ IL-4Rα^−/lox^ mice and their littermate control were cultured at 1 × 10^6^ cells/ml in RPMI medium for 18 or 36 hr, at 37°C in a humidified atmosphere containing 5% CO_2_, with 0 or 10 ng/ml rIL-4; then, cells were checked for survival and Foxp3 expression by flow cytometry.

### In vitro Treg cell conversion assay

CD4^+^ T cells were enriched from a pool of naïve spleens and MLN cells by using EasySep Mouse T Cell Isolation Kit (Stemcell Technologies, Vancouver, Canada, Catalogue no. 19852A) as per the manufacturer’s instruction, and then, the CD4^+^ CD25^−^ population was sorted by using BD FACSAria III cell sorter (BD Biosciences). Sorted CD4^+^ CD25^−^ cells (2.5 × 10^6^ cells/ml) were cultured with plate-bound anti-CD3 (10 μg/ml) in the presence of soluble anti-CD28 (2 μg/ml) and different concentrations of TGFβ (0, 1, or 2 ng/ml) (BD Pharmingen) in 96-well, flat-bottomed plates in triplicates. After 3 d, the iTreg cells were analyzed by flow cytometry for CD25 and Foxp3 expression.

### Serum analyses

#### Cytokines and IgE

Serum cytokines (IL-4 and TNF-α) and total IgE titer were measured as follows. Blood was collected in serum separator tubes (BD Biosciences) and centrifuged at 8,000 *g* for 10 min at 4°C to separate serum. The Nunc MicroWell 96-Well Microplates (Thermo Fisher Scientific) were coated with 0.5 ng/ml capture antibody in PBS and incubated overnight at 4°C. Plates were washed and blocked with 2% (w/v) milk powder for 2 hr at 37°C, and samples were loaded and incubated overnight at 4°C. Detecting, biotin-labeled antibodies were added and incubated for 2 hr at 37°C, and then, avidin-horseradish peroxidase or avidin-alkaline phosphatase was added and incubated for 1 hr at 37°C. Plates were developed by adding TMB Microwell Peroxidase Substrate (KPL, Gaithersburg, MD, US) or *p*-nitrophenyl phosphate disodium salt hexahydrate (Sigma), respectively. The absorbance was read at 450 or 405 nm using a VersaMax microplate spectrophotometer (Molecular Devices, Sunnyvale, CA, US).

#### Liver enzymes

Hepatocellular damage was assessed by measuring the serum level of AST at the National Health Laboratory Service of South Africa (Cape Town).

### Histology

#### *Sm* infection

Liver and gut tissues were fixed in 4% (v/v) formaldehyde in PBS, embedded in wax, and processed, and 5–7 μm sections were stained with HE. Micrographs of liver and gut granuloma were captured using Nikon Eclipse 90i (Nikon, Minato, Tokyo). A granuloma diameter of 20–50 granulomas per animal was determined using Nikon NIS-Elements imaging software (Nikon Corporation). For fibrosis assessment, tissue sections were stained with chromotrope 2R and CAB and counterstained with Wegert’s hematoxylin for collagen staining [23].

#### *Nb* infection

Lung tissues were fixed in 4% (v/v) formaldehyde in PBS, embedded in wax, and processed, and 5 μm sections were stained with periodic acid-Schiff reagent (PAS) to visualize mucus-producing goblet cells [52]. Micrographs of lung sections were captured using Nikon Eclipse 90i.

### Tissue homogenate for cytokine analysis

Livers were collected and homogenized in lysis buffer (PBS [pH 7.1], 0.1% Tween 20 [Merck], and 1% protease inhibitor cocktail [Sigma-Aldrich, St. Louis, MO, US, catalogue no. P8340]). Cytokines (IL-4, IL-5, IL-10, IL-13, IFN-γ, and IL-17, all from BD Pharmingen) were measured in the protein extracts by sandwich ELISA as described previously [23]. Cytokine values were normalized according to the protein content measured by Pierce BCA Protein Assay Kit (Thermo Fisher Scientific, catalogue no. 23225).

### Hydroxyproline quantification

Hydroxyproline content as a measure of collagen production was quantified using a modified protocol [53]. In brief, weighed liver samples were hydrolyzed overnight at 110°C in 6 M HCl and then filtered through Whatman filter papers. Filtrate was neutralized with 1% phenolphthalein and titrated against 10 M NaOH. An aliquot was mixed with isopropanol and added to a chloramine-T/citrate buffer solution (pH 6.0) (Sigma). Ehrlich’s reagent solution (25 g *p*-dimethyl-amino-benzaldehyde, 37.5 ml 60% perchloric acid) was added and measured at 570 nm using a VersaMax microplate spectrophotometer (Molecular Devices). Hydroxyproline levels were calculated by using 4-hydroxy-L-proline (Calbiochem, San Diego, CA, US) as standard, and results were expressed as μg hydroxyproline per weight of liver tissue that contained 10^4^ eggs.

### Statistics

Statistical analysis was conducted using GraphPad Prism 4 software (http://www.prism-software.com). Data were calculated as the mean ± SD. Statistical significance was determined using the unpaired Student *t* test and one-way ANOVA with Bonferroni’s posttest, defining differences to IL-4Rα^−/lox^ mice as significant (*, *P* ≤ 0.05; **, *P* ≤ 0.01; ***, *P* ≤ 0.001).

## Supporting information

S1 Data Underlying data for main and supplementary figures. (XLSX)

S1 Fig Generation of Foxp3Cre^−/+^ IL-4Rα^−/lox^ BALB/c mice. Flow charts illustrating the generation of Foxp3Cre^−/+^ IL-4Rα^−/lox^ BALB/c mice. Either male (A) or female (B) Foxp3Cre^−/+^ mice can be used to start the generation of male and female Foxp3Cre^−/+^ IL-4Rα^−/lox^ mice and their littermate controls. The breeding scheme followed in the present study is highlighted in red. The gene genotype is indicated above the drawn chromosome, and the number of the chromosome is indicated below. Cre, cyclic recombinase; D, deleted; F, floxed; Foxp3, forkhead box P3; IL-4Rα, interleukin-4 receptor alpha; N, no modifications (wild type); T, transgenic (Cre transgene).
(TIF)

S2 Fig STAT6 phosphorylation of male and female Foxp3^cre^ IL-4Rα^−/lox^ mice upon rIL-4 stimulation. Cells pooled from spleen and MLNs from naïve male and female IL-4Rα^−/lox^, Foxp3^cre^ IL-4Rα^−/lox^, and IL-4Rα^−/−^ mice were cultured for 1 hr in 0 or 10 ng/ml rIL-4, and STAT6 phosphorylation was then analyzed by flow cytometry. (A) Gating strategy for identifying CD19^+^ B cells, Foxp3^−^ T cells, and Foxp3^+^ Treg cell populations for calculating p-STAT6 expression. (B) Flow cytometry analysis of STAT6 phosphorylation at baseline in cell populations indicated in (A). (C) Flow cytometry analysis of STAT6 phosphorylation after rIL-4 stimulation for 1 hr in cell populations indicated in (A). (D) Formula for calculating the variation of STAT6 phosphorylation at baseline and after rIL-4 stimulation. (E) Variation in the level of STAT6 phosphorylation, before and after rIL-4 stimulation, calculated by the formula in (D). Results are representative of two independent experiments with 3–4 mice/group. Data are expressed as mean ± S.E.M. ns, *P* > 0.05; * *P* < 0.05, ** *P* < 0.001, *** *P* < 0.0001 by two-tailed unpaired Student *t* test. Underlying data can be found in S1 Data. CD3, cluster of differentiation 3; CD4, cluster of differentiation 4; CD19, cluster of differentiation 19; Foxp3, forkhead box P3; FSC, forward scatter; GMFI, geometric mean fluorescence intensity; IL-4Rα, interleukin-4 receptor alpha; MLN, mesenteric lymph node; ns, not significant; p-STAT6, phosphorylated STAT6; rIL-4, recombinant interleukin-4; SSC, side scatter; STAT6, signal transducer and activator of transcription 6; Treg, regulatory T.
(TIF)

S3 Fig Deletion of IL-4Rα on Foxp3^+^ Treg cells neither alters Foxp3^+^ Treg cell compartment nor breaks the tolerance under a steady state in either male or female Foxp3^cre^ IL-4Rα^−/lox^ mice. (A) Frequency of CD4^+^ Foxp3^+^ T cells from spleen, lung, MLN, and thymus of naïve male and female IL-4Rα^−/lox^ and Foxp3^cre^ IL-4Rα^−/lox^ mice. (B) Body weight of naïve male and female IL-4Rα^−/lox^ and Foxp3^cre^ IL-4Rα^−/lox^ mice. (C) Organ weights of naïve male and female mice. (D) Total cell number of spleen, liver, lung, MLN, and thymus of naïve male and female mice. (E) Frequency of CD3^+^, (F) CD3^+^ CD8^+^, and (G) CD3^+^ CD4^+^ T cells from organs of mice as in (D). (H) Frequency of CD19^+^ B cells in spleen, lung, and MLN of naïve male and female mice. (I) Frequency of DP and DN T cells in the thymus of naïve male and female mice. (J) Serum analysis of naïve mice. (K) Analysis of liver function in naïve male and female mice. (L) Frequency of IFN-γ-, IL-4-, IL-10-, and IL-13-expressing CD4^+^ T cells. MLN cells from naïve male and female mice were restimulated with PMA/Ionomycin in the presence of monensin, after which CD4^+^ T cells stained intracellularly for indicated cytokines. Results are representative of two independent experiments with 7–9 mice/group. Data are expressed as mean ± S.E.M. ns, *P* > 0.05; * *P* < 0.05, ** *P* < 0.001, *** *P* < 0.0001 by two-tailed unpaired Student *t* test. Underlying data can be found in S1 Data. CD3, cluster of differentiation 3; CD4, cluster of differentiation 4; CD8, cluster of differentiation 8; CD19, cluster of differentiation 19; DP, double positive; DN, double negative; Foxp3, forkhead box P3; IFN-γ, interferon gamma; IgE, immunoglobulin E; IL-4, interleukin-4; IL-10, interleukin-10; IL-13, interleukin-13; IL-4Rα, interleukin-4 receptor alpha; MLN, mesenteric lymph node; ND, not detectable; ns, not significant; PMA, phorbol myristate acetate; SSC, side scatter; TNFα, tumor necrosis factor alpha; Treg, regulatory T.
(TIF)

S4 Fig IL-4Rα signaling is dispensable for Foxp3 Treg cell conversion in vitro but promotes the survival of and enhances expression of Foxp3 in CD4^+^ CD25^+^ T cells. (A) Representative flow cytometric analysis of the CD4^+^ CD25^−^ and CD4^+^ CD25^+^ cell populations before and after FACS of pooled cells from spleen and MLN of naïve IL-4Rα^−/lox^ and Foxp3^cre^ IL-4Rα^−/lox^ mice. (B) Representative flow cytometry of converted CD4^+^ CD25^+^ Foxp3^+^ Treg cells from CD4^+^ CD25^−^ T cells cultured with gradient concentration of TGFβ for 72 hr in presence of TCR stimuli. (C) Frequency of iTreg cells generated in vitro from (B). (D) CD4^+^ CD25^+^ T-cell survival in presence and absence or rIL-4 (10 ng/ml). Sorted CD4^+^ CD25^+^ T cells from naïve IL-4Rα^−/Lox^ and Foxp3^Cre^ IL-4Rα^−/Lox^ mice were cultured for 18 or 36 hr with or without rIL-4. (E) Frequency of CD25^+^ Foxp3^+^ T cells. (F) Representative histograms of Foxp3 expression by CD25^+^ Foxp3^+^ T cells 36 hr post rIL-4 stimulation with the mean values summarized in (G). Results are representative of four independent experiments with 5–7 mice/group. Data are expressed as mean ± S.E.M. NS, *P* > 0.05; * *P* < 0.05, ** *P* < 0.001, *** *P* < 0.0001 by two-tailed unpaired Student *t* test. Underlying data can be found in S1 Data. CD4, cluster of differentiation 4; CD25, cluster of differentiation 25; FACS, fluorescence-activated cell sorting; Foxp3, forkhead box P3; GMFI, geometric mean fluorescence intensity; IL-4Rα, interleukin-4 receptor alpha; iTreg, induced Treg; NS, not significant; rIL-4, recombinant interleukin-4; TCR, T-cell receptor; TGFβ, transforming growth factor beta; Treg, regulatory T.
(TIF)

S5 Fig IL-4Rα-mediated signaling on Foxp3^+^ Treg cell is required for Treg cell accumulation and effector T-cell control in male and female *Sm*-infected Foxp3^cre^ IL-4Rα^−/lox^ mice. Male and female IL-4Rα^−/lox^, Foxp3^cre^ IL-4Rα^−/lox^, and IL-4Rα^−/−^ mice were infected with *Sm* cercariae and euthanized 8 wk post infection. (A) Flow cytometry analysis of IL-4Rα expression by CD19^+^ B cell and (B) CD4^+^ Foxp3^+^ T cell in pooled spleen and MLN cells 8 wk post infection. (C) Frequency of CD4^+^ Foxp3^+^ T cells from the liver of male and female mice infected with *Sm* for 8 wk. (D) Foxp3 GMFI in CD4^+^ Foxp3^+^ T cells from (C). (E) Liver cytokine production 8 wk post infection. Livers from infected male and female mice were homogenized, and the levels of the indicated cytokines were detected by ELISA and normalized to mg of liver tissue. (F) Liver hydroxyproline content measured by colorimetry 8 wk post infection in male and female mice. (G) Frequency of CD4^+^ Foxp3^+^ T cells from the MLN of male and female mice infected with *Sm* for 8 wk. (H) Foxp3 GMFI in CD4^+^ Foxp3^+^ T cells from (G). (I) Frequency of CD4^+^ Ki-67^+^ cells within CD4^+^ Foxp3^−^ T-cell population in MLNs 8 wk post infection. (J) Frequency of CD3^+^ CD4^+^ CD44^+^ effector T cells in MLNs 8 wk post infection. (K) Frequency of cytokine-producing CD3^+^ CD4^+^ T cells, from MLN of male and female infected mice, after stimulation with PMA/Ionomycin in the presence of monensin. (L) Frequency of indicated transcription factor–expressing CD4^+^ T cells in the MLNs 8 wk post infection. (M) Frequency of cytokine-producing CD4^+^ GATA3^+^ T cells, from MLNs, after stimulation with PMA/Ionomycin in the presence of monensin. (N) Frequency of cytokine-producing CD4^+^ Foxp3^+^ T cells, from MLN, after stimulation with PMA/Ionomycin in the presence of monensin. (O) Gut hydroxyproline content 8 wk post infection in male and female mice. Results are representative of two independent experiments with 6–10 mice/group. Data are expressed as mean ± S.E.M. ns, *P* > 0.05; * *P* < 0.05, ** *P* < 0.001, *** *P* < 0.0001 by two-tailed unpaired Student *t* test. Underlying data can be found in S1 Data. Bcl-6, B cell lymphoma 6; CD3, cluster of differentiation 3; CD4, cluster of differentiation 4; CD19, cluster of differentiation 19; CD44, cluster of differentiation 44; Foxp3, forkhead box P3; GATA3, GATA binding protein 3; GMFI, geometric mean fluorescence intensity; IL-4, interleukin-4; IL-4Rα, interleukin-4 receptor alpha; IL-5, interleukin-5; IL-10, interleukin-10; IL-13, interleukin-13; IL-17, interleukin-17; IFN-γ, interferon gamma; MLN, mesenteric lymph node; ns, not significant; PMA, phorbol myristate acetate; RORγt, RAR-related orphan receptor gamma; *Sm*, *S*. *mansoni*; T-bet; T-box transcription factor; Treg, regulatory T.
(TIF)

S6 Fig Gating strategies. (A) Transcription factor–expressing CD4^+^ T cells. (B) Cytokine-producing CD4^+^ GATA3^+^ and CD4^+^ Foxp3^+^ T cells. Bcl-6, B cell lymphoma 6; CD3, cluster of differentiation 3; CD4, cluster of differentiation 4; FMO, fluorescence minus one; Foxp3, forkhead box P3; FSC, forward scatter; GATA3, GATA binding protein 3; IL-4, interleukin-4; IL-10, interleukin-10; IL-13, interleukin-13; RORγt, RAR-related orphan receptor gamma; T-bet; T-box transcription factor.
(TIF)

S7 Fig Cre expression has neither a major impact on Foxp3^+^ Treg cell compartment nor immune responses in our Foxp3^cre^ transgenic mice. IL-4Rα^+/+^ and Foxp3^cre^ IL-4Rα^+/+^ mice were infected with 100 *Sm* cercariae and euthanized 8 wk after, and Foxp3^+^ Treg cell compartment was analyzed in liver and MLN. (A) Flow cytometry analysis of IL-4Rα expression by CD19^+^ B cell and CD4^+^ Foxp3^+^ T cell in pooled spleen and MLN cells 8 wk post infection. (B) Representative flow cytometry of CD4^+^ Foxp3^+^ T cells in the liver. (C) Frequency of CD4^+^ Foxp3^+^ T cells from (A). (D) Representative histogram of Foxp3 expression by CD4^+^ Foxp3^+^ T cells in the liver. (E) Foxp3 GMFI in CD4^+^ Foxp3^+^ T cells from (D). (F) Representative histogram of GATA3 expression by CD4^+^ Foxp3^+^ T cells in the liver 8 wk post infection with the mean values summarized in (G). (H) Representative flow cytometry of CD4^+^ Foxp3^+^ T cells in the MLN. (I) Frequency of CD4^+^ Foxp3^+^ T cells from (H). (J) Representative histogram of Foxp3 expression by CD4^+^ Foxp3^+^ T cells in MLN. (K) Foxp3 GMFI in CD4^+^ Foxp3^+^ T cells from (J). (L) Frequency of CD4^+^ GATA3^+^ T cells in MLN 8 wk post infection. (M) Liver cytokine production 8 wk post infection. Livers from infected mice were homogenized, and the levels of the indicated cytokines were detected by ELISA and normalized to mg of liver tissue. Results are representative of two independent experiments with 6–8 mice/group. Data are expressed as mean ± S.E.M. NS, *P* > 0.05; * *P* < 0.05, ** *P* < 0.001, *** *P* < 0.0001 by two-tailed unpaired Student *t* test. Underlying data can be found in S1 Data. CD4, cluster of differentiation 4; CD19, cluster of differentiation 19; Cre, cyclic recombinase; Foxp3, forkhead box P3; GATA3, GATA binding protein 3; GMFI, geometric mean fluorescence intensity; IFN-γ, interferon gamma; IL-4, interleukin-4; IL-4Rα, interleukin-4 receptor alpha; IL-5, interleukin-5; IL-10, interleukin-10; IL-13, interleukin-13; IL-17, interleukin-17; MLN, mesenteric lymph node; NS, not significant; Treg, regulatory T.
(TIF)

S8 Fig Cre expression does not induce exaggerated fibrogranulomatous inflammation during experimental *Sm* infection. (A) Representative HE staining of liver sections from IL-4Rα^−/Lox^ and Foxp3^Cre^ IL-4Rα^−/Lox^ mice infected with *Sm* for 8 wk (original magnification 100×). (B) Liver granuloma size. Granuloma size was determined from (A) by using a computerized morphometric analysis program (NIS elements by NIKON) by measuring 100 granulomas/group. (C) Representative CAB-stained liver sections from *Sm*-infected mice (original magnification 100×). (D) Liver hydroxyproline content measured by colorimetry 8 wk post infection. (E) Representative HE staining of gut sections from mice infected with *Sm* for 8 wk (original magnification 100×). (F) Representative CAB-stained gut sections 8 wk post infection (original magnification 100×). (G) Gut hydroxyproline content 8 wk post infection. Results are representative of two independent experiments with 6–8 mice/group. Data are expressed as mean ± S.E.M. NS, *P* > 0.05; * *P* < 0.05, ** *P* < 0.001, *** *P* < 0.0001 by two-tailed unpaired Student *t* test. Underlying data can be found in S1 Data. CAB, chromotrope aniline blue; Cre, cyclic recombinase; Foxp3, forkhead box P3; HE, hematoxylin–eosin; IL-4Rα, interleukin-4 receptor alpha; NS, not significant; *Sm*, *S*. *mansoni*.
(TIF)

S9 Fig IL-4Rα signaling on Foxp3^+^ Treg cells is required to control worm-driven lung inflammation during *Nb* infection. (A) Representative PAS staining of mucus-producing goblet cells, with lower (20×, top) and higher (200×) magnifications, in the lung tissues 9 d post infection of IL-4Rα^−/lox^ and Foxp3^cre^ IL-4Rα^−/lox^ mice with 500 L3 larvae of *Nb*. (B) Quantification of PAS^+^ bronchi/lung/mouse. (C) Representative of Foxp3^+^ Treg cell infiltration within the lung alveoli 9 d post *Nb* infection (original magnification 200×). Thin arrows point to inner and outer Foxp3^+^ cells. (D) Ratio of inner to outer Foxp3^+^ Treg cells per alveoli. Results are representative of two independent experiments with 5–7 mice/group. Data are expressed as mean ± S.E.M. NS, *P* > 0.05; * *P* < 0.05, ** *P* < 0.001, *** *P* < 0.0001 by two-tailed unpaired Student *t* test. Underlying data can be found in S1 Data. Foxp3, forkhead box P3; IL-4Rα, interleukin-4 receptor alpha; *Nb*, *N*. *brasiliensis*; NS, not significant; PAS, periodic acid-Schiff reagent; Treg, regulatory T.
(TIF)

S10 Fig IL-4Rα-mediated signaling on Foxp3^+^ Treg cells is required for eTreg cell generation and maintenance or up-regulation of CXCR3 in the MLN during experimental *Sm* infection. (A) Frequency of central CD4^+^ Foxp3^+^ Treg cells (left) and (B) effector CD4^+^ Foxp3^+^ Treg cells. (C) Frequency of CXCR3^+^ population within the effector CD4^+^ Foxp3^+^ Treg cells. (D) CXCR3 GMFI within the effector CD4^+^ Foxp3^+^ Treg cells. Results pooled from two independent experiments with 3–4 mice/group. Data are expressed as mean ± S.E.M. NS, *P* > 0.05; * *P* < 0.05, ** *P* < 0.001, *** *P* < 0.0001 by two-tailed unpaired Student *t* test. Underlying data can be found in S1 Data. cTreg, central Foxp3^+^ regulatory T; CXCR3, C-X-C motif chemokine receptor 3, eTreg, effector regulatory T; Foxp3, forkhead box P3; GMFI, geometric mean fluorescence intensity; IL-4Rα, interleukin-4 receptor alpha; MLN, mesenteric lymph node; NS, not significant; Treg, regulatory T.
(TIF)

## Abbreviations

AST: aspartate aminotransferase
Bcl-2: B cell lymphoma 2
Bcl-6: B cell lymphoma 6
CAB: chromotrope aniline blue
CD3: cluster of differentiation 3
CD4: cluster of differentiation 4
CD19: cluster of differentiation 19
CD25: cluster of differentiation 25
CD44: cluster of differentiation 44
Cre: cyclic recombinase
cTreg: central Foxp3^+^ Treg
CXCR3: C-X-C motif chemokine receptor 3
Ed: efficiency of deletion
eTreg: effector Foxp3^+^ Treg
FMO: fluorescence minus one
Foxp3: forkhead box P3
FSC: forward scatter
GATA3: GATA binding protein 3
GMFI: geometric mean fluorescence intensity
HE: hematoxylin–eosin
hLN: hepatic lymph node
iFCS: inactivated fetal calf serum
IFN-γ: interferon gamma
IgE: immunoglobulin E
IL-2: interleukin-2
IL-4: interleukin-4
IL-5: interleukin-5
IL-6: interleukin-6
IL-10: interleukin-10
IL-12: interleukin-12
IL-13: interleukin-13
IL-15: interleukin-15
IL-17: interleukin-17
IL-4Rα: IL-4 receptor alpha
IMDM: Iscove’s Modified Dulbecco’s Medium
iTreg: induced Treg
loxP: locus of X-over P1
MLN: mesenteric lymph node
Nb: *N*. *brasiliensis*
ND: not detectable
NS: not significant
p-STAT6: phosphorylated STAT6
qPCR: quantitative real-time PCR
PAS: periodic acid-Schiff reagent
PMA: phorbol myristate acetate
rIL-4: recombinant IL-4
RORγt: RAR-related orphan receptor gamma
SSC: side scatter
STAT6: signal transducer and activator of transcription 6
T-bet: T-box transcription factor
TGF-β1: transforming growth factor beta 1
Th1: T helper 1
Th2: T helper 2
Th17: T helper 17
TNF-α: tumor necrosis factor alpha
Treg: regulatory T
