# Supplementary figures and images for: The Foxp3+ regulatory T-cell population requires IL-4Rα signaling to control inflammation during helminth infections
Source: PLoS Biol. 2018 Oct 31;16(10):e2005850. doi: 10.1371/journal.pbio.2005850 (PMC6231676; doi:10.1371/journal.pbio.2005850)

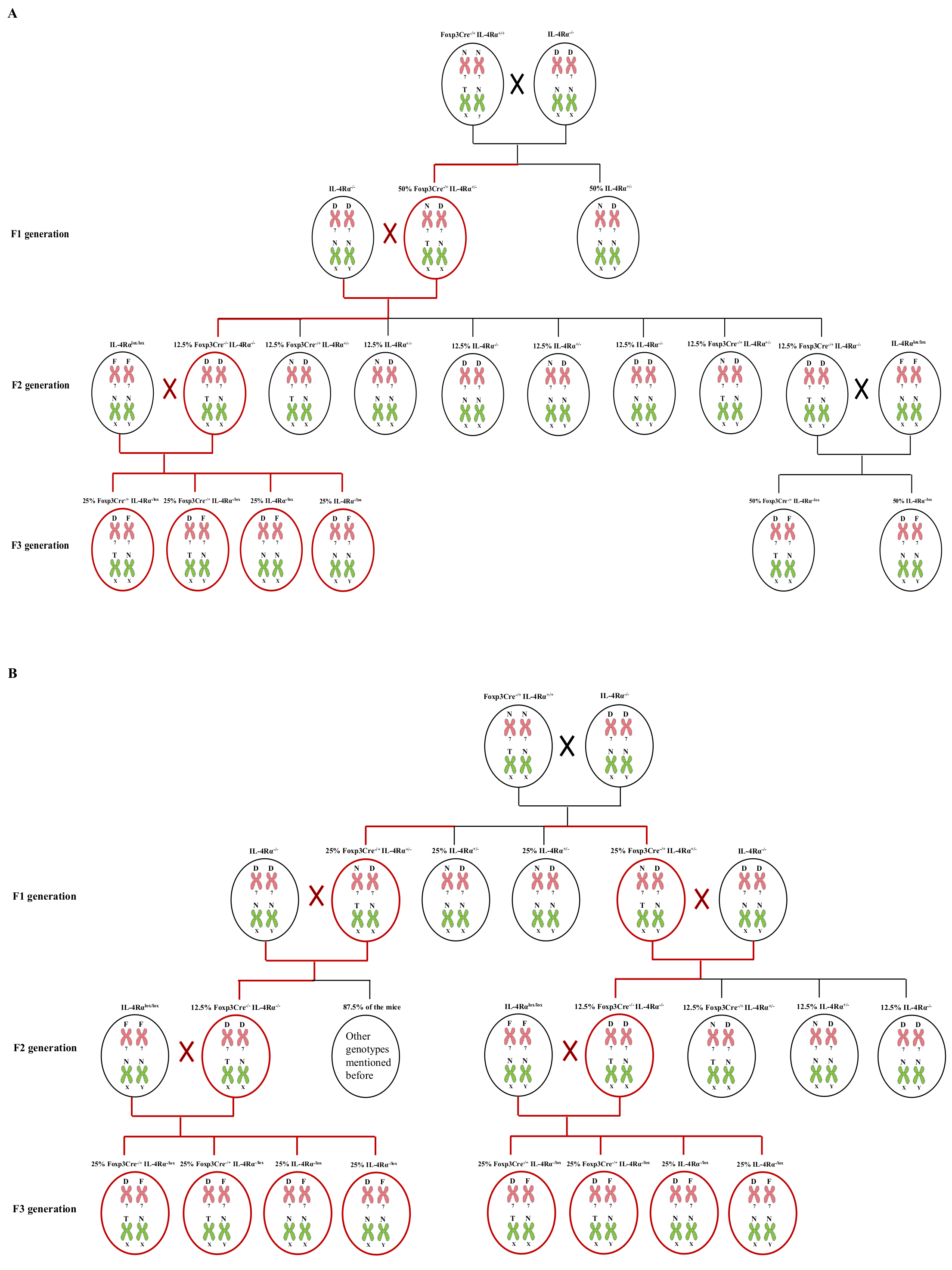

Supplement: S1 Fig — Flow charts illustrating the generation of Foxp3Cre−/+ IL-4Rα−/lox BALB/c mice. Either male (A) or female (B) Foxp3Cre−/+ mice can be used to start the generation of male and female Foxp3Cre−/+ IL-4Rα−/lox mice and their littermate controls. The breeding scheme followed in the present study is highlighted in red. The gene genotype is indicated above the drawn chromosome, and the number of the chromosome is indicated below. Cre, cyclic recombinase; D, deleted; F, floxed; Foxp3, forkhead box P3; IL-4Rα, interleukin-4 receptor alpha; N, no modifications (wild type); T, transgenic (Cre transgene). (TIF) [file pbio.2005850.s002.tif]

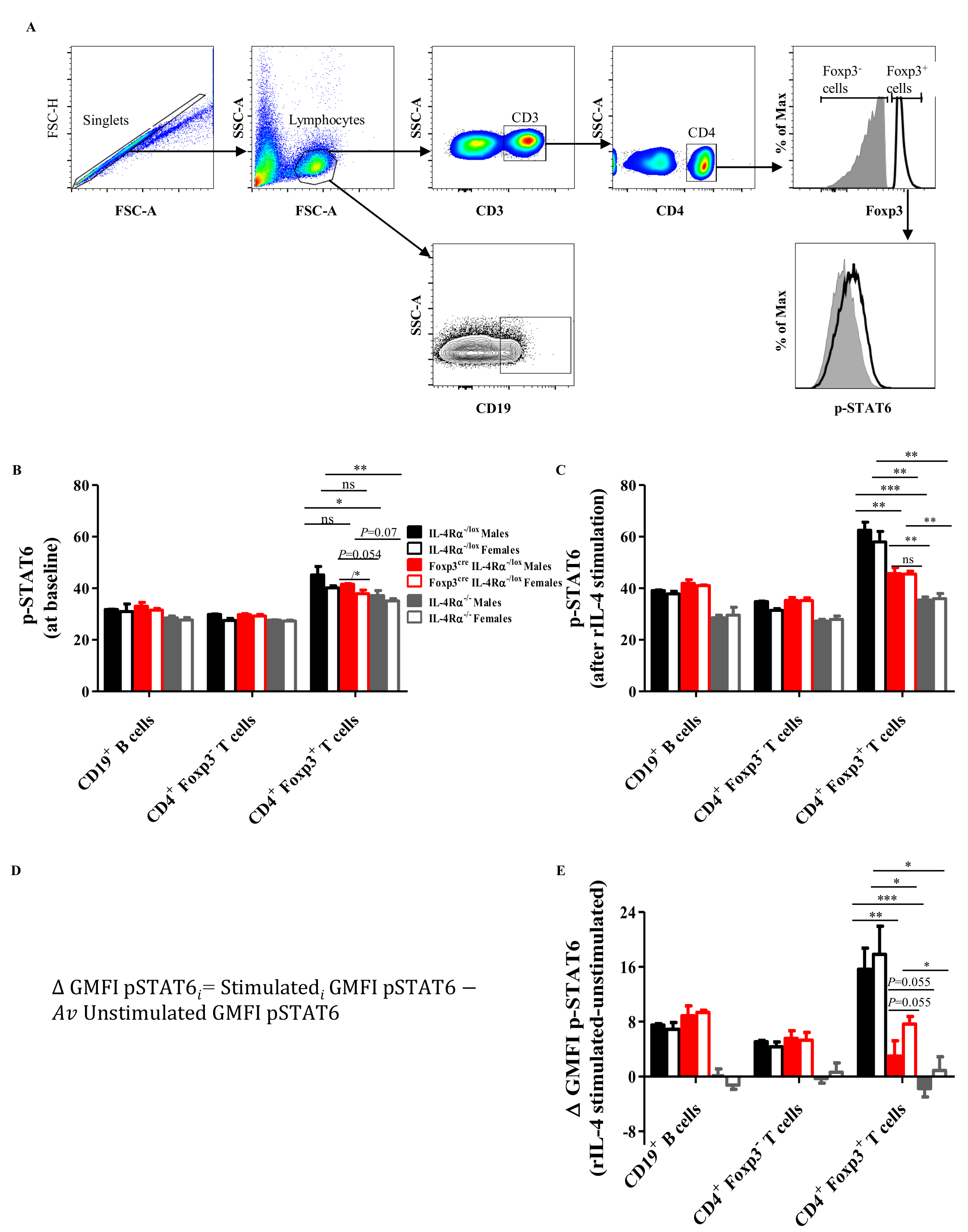

Supplement: S2 Fig — Cells pooled from spleen and MLNs from naïve male and female IL-4Rα−/lox, Foxp3cre IL-4Rα−/lox, and IL-4Rα−/− mice were cultured for 1 hr in 0 or 10 ng/ml rIL-4, and STAT6 phosphorylation was then analyzed by flow cytometry. (A) Gating strategy for identifying CD19+ B cells, Foxp3− T cells, and Foxp3+ Treg cell populations for calculating p-STAT6 expression. (B) Flow cytometry analysis of STAT6 phosphorylation at baseline in cell populations indicated in (A). (C) Flow cytometry analysis of STAT6 phosphorylation after rIL-4 stimulation for 1 hr in cell populations indicated in (A). (D) Formula for calculating the variation of STAT6 phosphorylation at baseline and after rIL-4 stimulation. (E) Variation in the level of STAT6 phosphorylation, before and after rIL-4 stimulation, calculated by the formula in (D). Results are representative of two independent experiments with 3–4 mice/group. Data are expressed as mean ± S.E.M. ns, P > 0.05; * P < 0.05, ** P < 0.001, *** P < 0.0001 by two-tailed unpaired Student t test. Underlying data can be found in S1 Data. CD3, cluster of differentiation 3; CD4, cluster of differentiation 4; CD19, cluster of differentiation 19; Foxp3, forkhead box P3; FSC, forward scatter; GMFI, geometric mean fluorescence intensity; IL-4Rα, interleukin-4 receptor alpha; MLN, mesenteric lymph node; ns, not significant; p-STAT6, phosphorylated STAT6; rIL-4, recombinant interleukin-4; SSC, side scatter; STAT6, signal transducer and activator of transcription 6; Treg, regulatory T. (TIF) [file pbio.2005850.s003.tif]

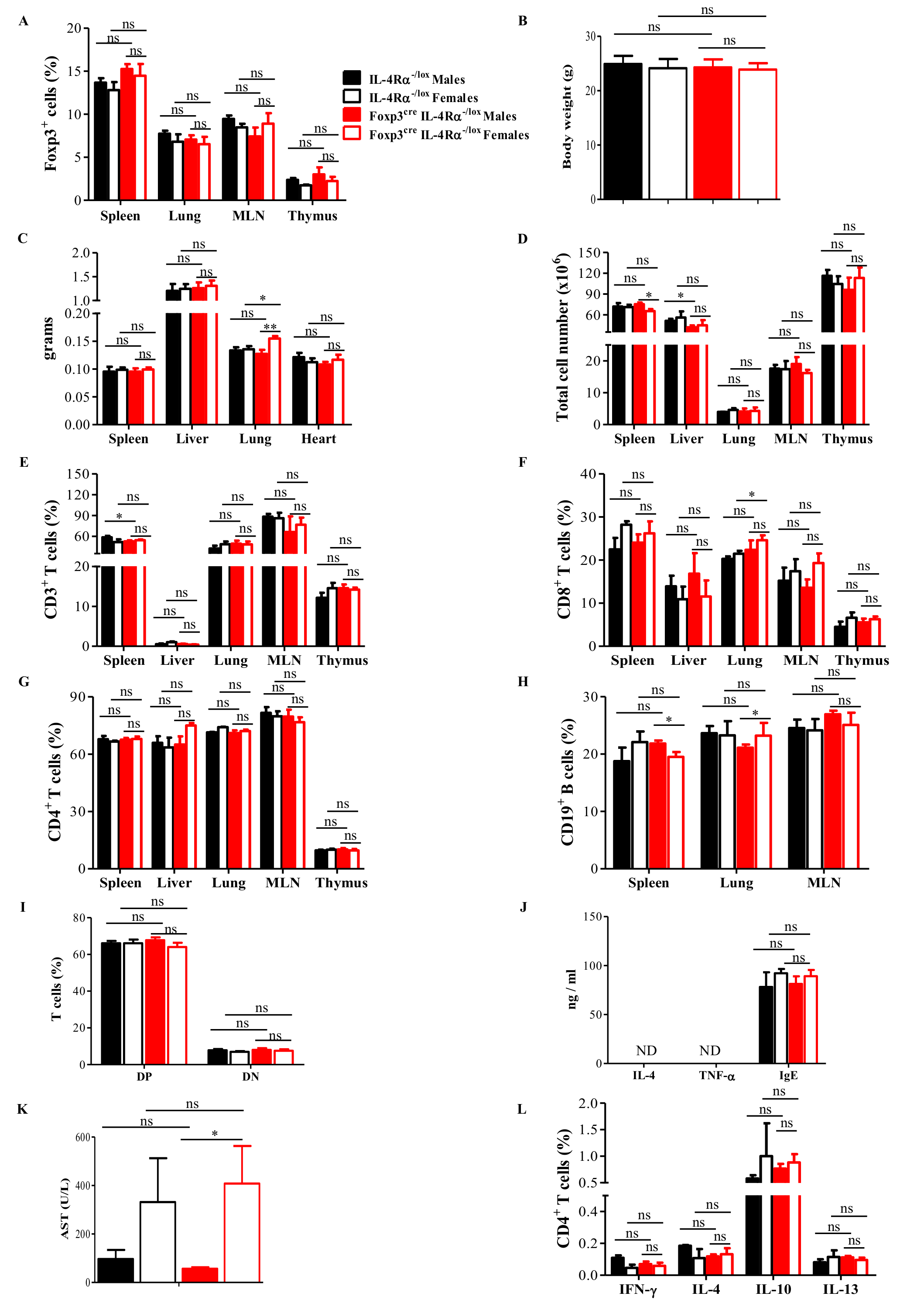

Supplement: S3 Fig — (A) Frequency of CD4+ Foxp3+ T cells from spleen, lung, MLN, and thymus of naïve male and female IL-4Rα−/lox and Foxp3cre IL-4Rα−/lox mice. (B) Body weight of naïve male and female IL-4Rα−/lox and Foxp3cre IL-4Rα−/lox mice. (C) Organ weights of naïve male and female mice. (D) Total cell number of spleen, liver, lung, MLN, and thymus of naïve male and female mice. (E) Frequency of CD3+, (F) CD3+ CD8+, and (G) CD3+ CD4+ T cells from organs of mice as in (D). (H) Frequency of CD19+ B cells in spleen, lung, and MLN of naïve male and female mice. (I) Frequency of DP and DN T cells in the thymus of naïve male and female mice. (J) Serum analysis of naïve mice. (K) Analysis of liver function in naïve male and female mice. (L) Frequency of IFN-γ-, IL-4-, IL-10-, and IL-13-expressing CD4+ T cells. MLN cells from naïve male and female mice were restimulated with PMA/Ionomycin in the presence of monensin, after which CD4+ T cells stained intracellularly for indicated cytokines. Results are representative of two independent experiments with 7–9 mice/group. Data are expressed as mean ± S.E.M. ns, P > 0.05; * P < 0.05, ** P < 0.001, *** P < 0.0001 by two-tailed unpaired Student t test. Underlying data can be found in S1 Data. CD3, cluster of differentiation 3; CD4, cluster of differentiation 4; CD8, cluster of differentiation 8; CD19, cluster of differentiation 19; DP, double positive; DN, double negative; Foxp3, forkhead box P3; IFN-γ, interferon gamma; IgE, immunoglobulin E; IL-4, interleukin-4; IL-10, interleukin-10; IL-13, interleukin-13; IL-4Rα, interleukin-4 receptor alpha; MLN, mesenteric lymph node; ND, not detectable; ns, not significant; PMA, phorbol myristate acetate; SSC, side scatter; TNFα, tumor necrosis factor alpha; Treg, regulatory T. (TIF) [file pbio.2005850.s004.tif]

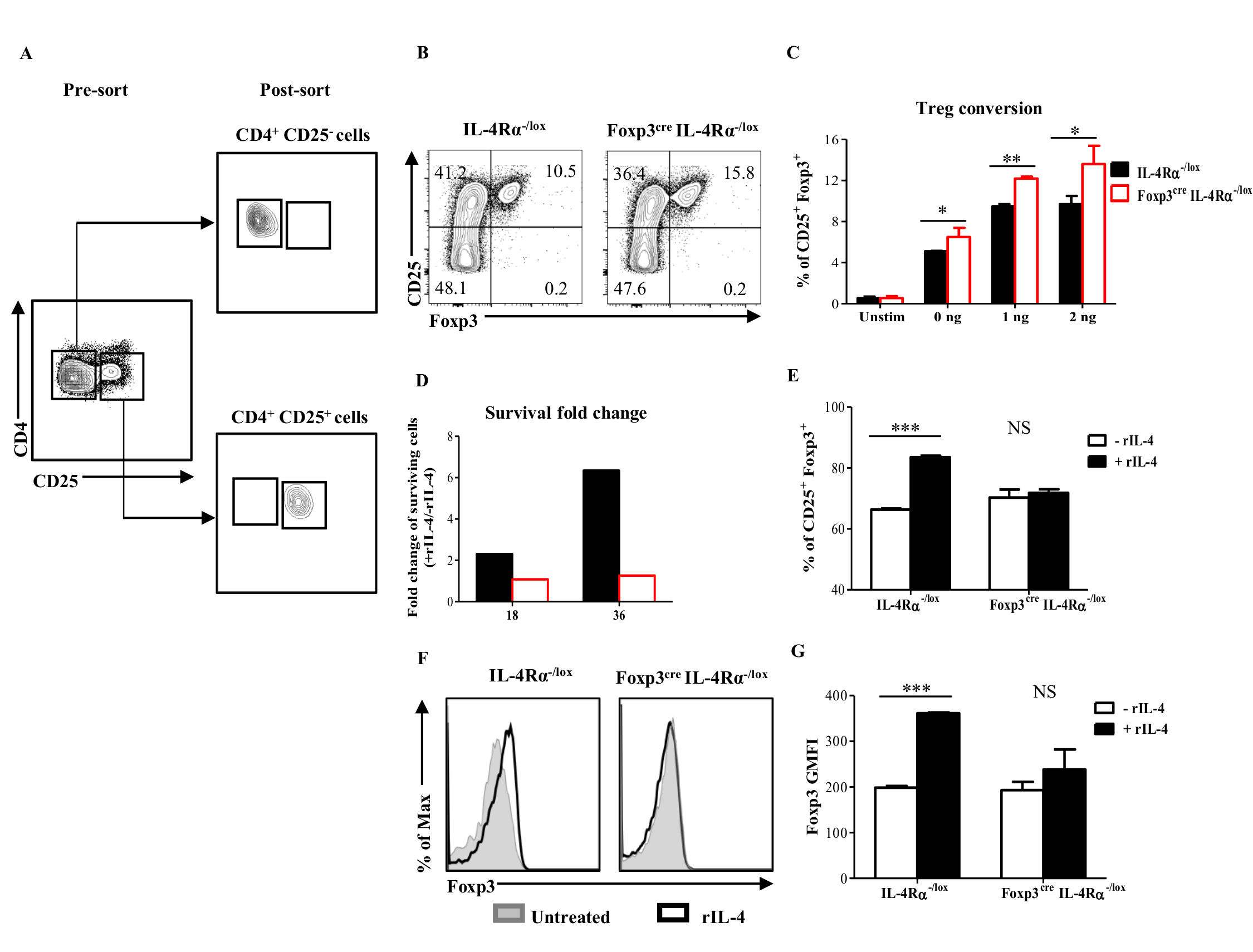

Supplement: S4 Fig — (A) Representative flow cytometric analysis of the CD4+ CD25− and CD4+ CD25+ cell populations before and after FACS of pooled cells from spleen and MLN of naïve IL-4Rα−/lox and Foxp3cre IL-4Rα−/lox mice. (B) Representative flow cytometry of converted CD4+ CD25+ Foxp3+ Treg cells from CD4+ CD25− T cells cultured with gradient concentration of TGFβ for 72 hr in presence of TCR stimuli. (C) Frequency of iTreg cells generated in vitro from (B). (D) CD4+ CD25+ T-cell survival in presence and absence or rIL-4 (10 ng/ml). Sorted CD4+ CD25+ T cells from naïve IL-4Rα−/Lox and Foxp3Cre IL-4Rα−/Lox mice were cultured for 18 or 36 hr with or without rIL-4. (E) Frequency of CD25+ Foxp3+ T cells. (F) Representative histograms of Foxp3 expression by CD25+ Foxp3+ T cells 36 hr post rIL-4 stimulation with the mean values summarized in (G). Results are representative of four independent experiments with 5–7 mice/group. Data are expressed as mean ± S.E.M. NS, P > 0.05; * P < 0.05, ** P < 0.001, *** P < 0.0001 by two-tailed unpaired Student t test. Underlying data can be found in S1 Data. CD4, cluster of differentiation 4; CD25, cluster of differentiation 25; FACS, fluorescence-activated cell sorting; Foxp3, forkhead box P3; GMFI, geometric mean fluorescence intensity; IL-4Rα, interleukin-4 receptor alpha; iTreg, induced Treg; NS, not significant; rIL-4, recombinant interleukin-4; TCR, T-cell receptor; TGFβ, transforming growth factor beta; Treg, regulatory T. (TIF) [file pbio.2005850.s005.tif]

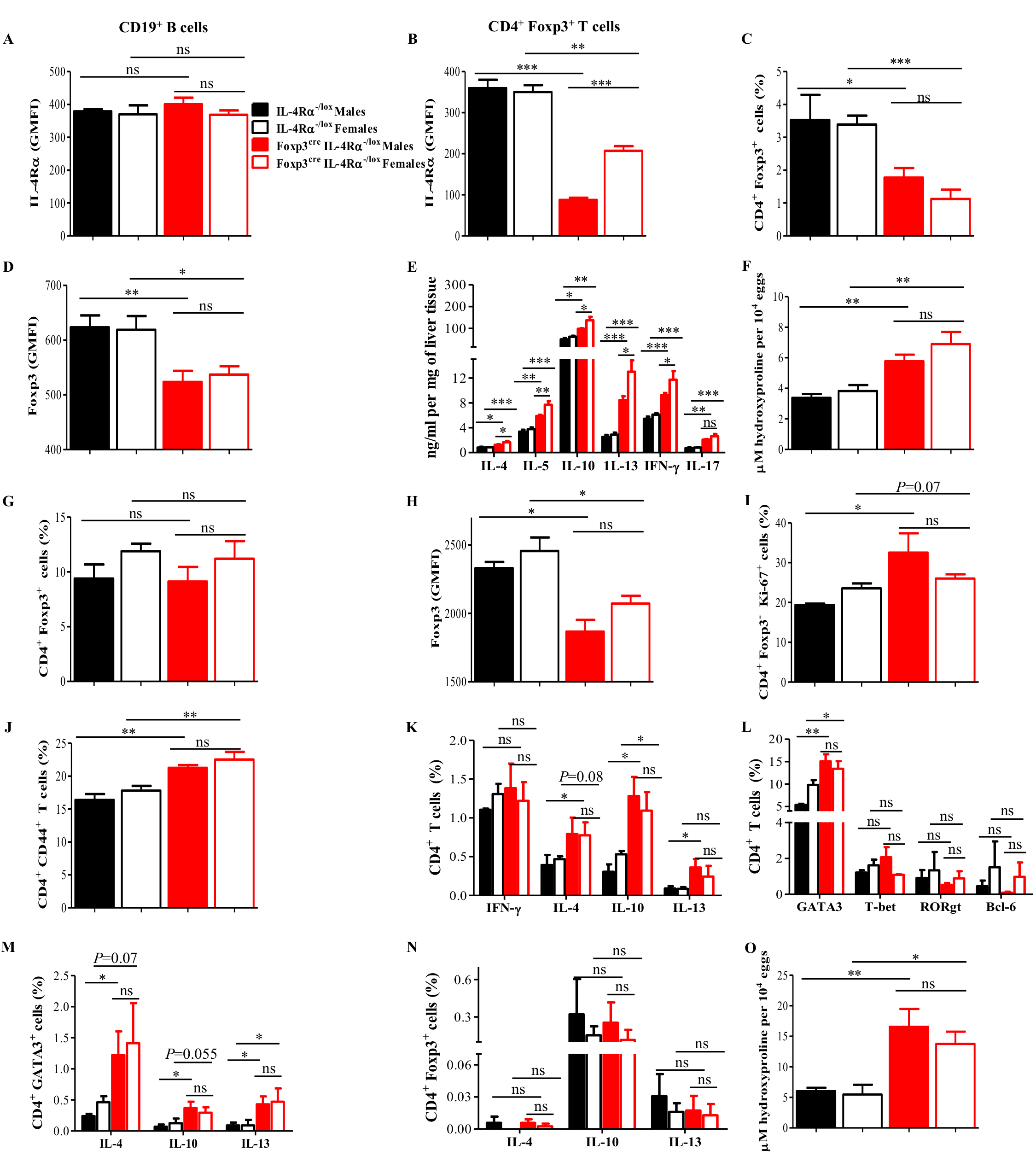

Supplement: S5 Fig — Male and female IL-4Rα−/lox, Foxp3cre IL-4Rα−/lox, and IL-4Rα−/− mice were infected with Sm cercariae and euthanized 8 wk post infection. (A) Flow cytometry analysis of IL-4Rα expression by CD19+ B cell and (B) CD4+ Foxp3+ T cell in pooled spleen and MLN cells 8 wk post infection. (C) Frequency of CD4+ Foxp3+ T cells from the liver of male and female mice infected with Sm for 8 wk. (D) Foxp3 GMFI in CD4+ Foxp3+ T cells from (C). (E) Liver cytokine production 8 wk post infection. Livers from infected male and female mice were homogenized, and the levels of the indicated cytokines were detected by ELISA and normalized to mg of liver tissue. (F) Liver hydroxyproline content measured by colorimetry 8 wk post infection in male and female mice. (G) Frequency of CD4+ Foxp3+ T cells from the MLN of male and female mice infected with Sm for 8 wk. (H) Foxp3 GMFI in CD4+ Foxp3+ T cells from (G). (I) Frequency of CD4+ Ki-67+ cells within CD4+ Foxp3− T-cell population in MLNs 8 wk post infection. (J) Frequency of CD3+ CD4+ CD44+ effector T cells in MLNs 8 wk post infection. (K) Frequency of cytokine-producing CD3+ CD4+ T cells, from MLN of male and female infected mice, after stimulation with PMA/Ionomycin in the presence of monensin. (L) Frequency of indicated transcription factor–expressing CD4+ T cells in the MLNs 8 wk post infection. (M) Frequency of cytokine-producing CD4+ GATA3+ T cells, from MLNs, after stimulation with PMA/Ionomycin in the presence of monensin. (N) Frequency of cytokine-producing CD4+ Foxp3+ T cells, from MLN, after stimulation with PMA/Ionomycin in the presence of monensin. (O) Gut hydroxyproline content 8 wk post infection in male and female mice. Results are representative of two independent experiments with 6–10 mice/group. Data are expressed as mean ± S.E.M. ns, P > 0.05; * P < 0.05, ** P < 0.001, *** P < 0.0001 by two-tailed unpaired Student t test. Underlying data can be found in S1 Data. Bcl-6, B cell lymphoma 6; CD3, cluster of differentiation 3 [file pbio.2005850.s006.tif]

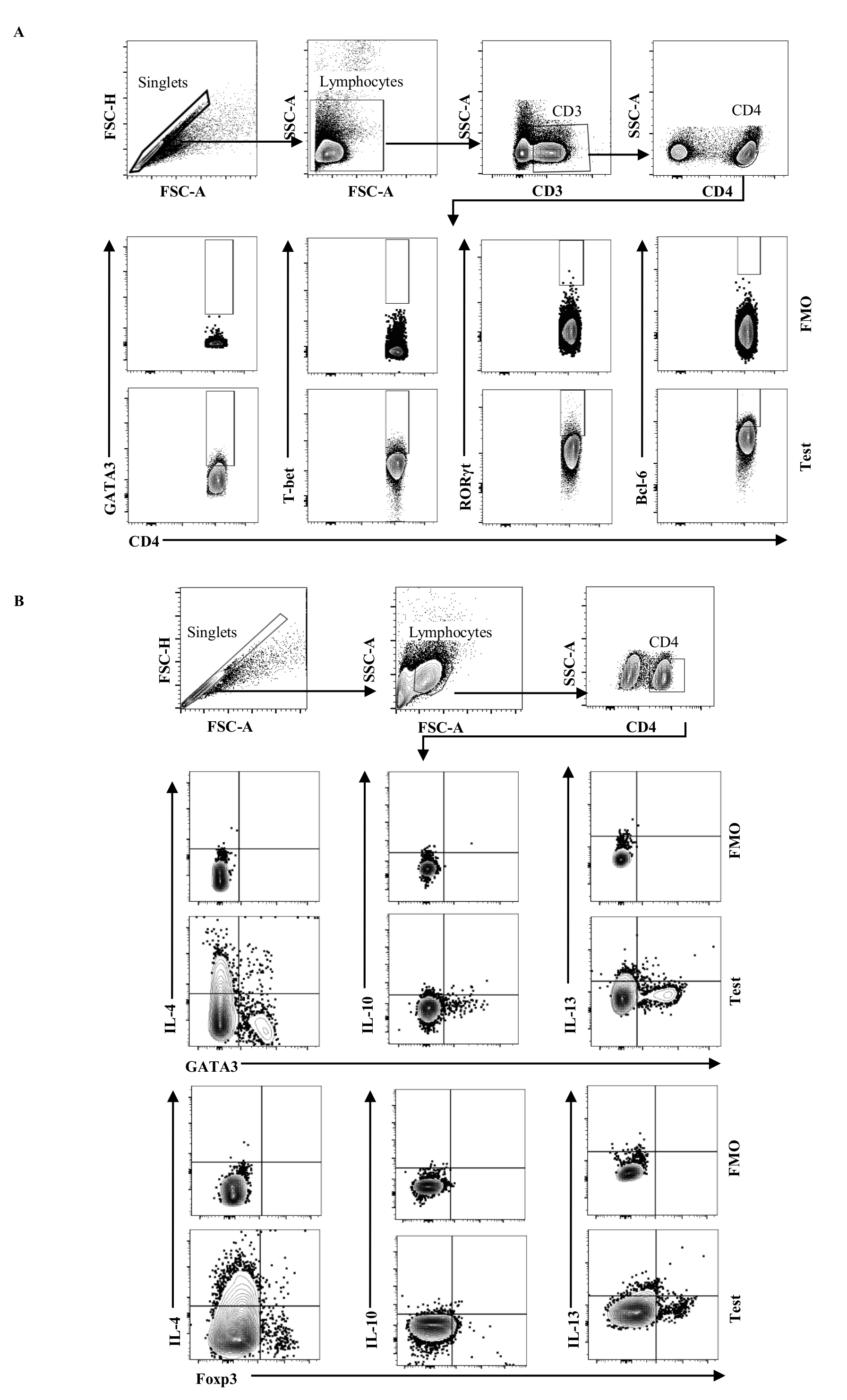

Supplement: S6 Fig — (A) Transcription factor–expressing CD4+ T cells. (B) Cytokine-producing CD4+ GATA3+ and CD4+ Foxp3+ T cells. Bcl-6, B cell lymphoma 6; CD3, cluster of differentiation 3; CD4, cluster of differentiation 4; FMO, fluorescence minus one; Foxp3, forkhead box P3; FSC, forward scatter; GATA3, GATA binding protein 3; IL-4, interleukin-4; IL-10, interleukin-10; IL-13, interleukin-13; RORγt, RAR-related orphan receptor gamma; T-bet; T-box transcription factor. (TIF) [file pbio.2005850.s007.tif]

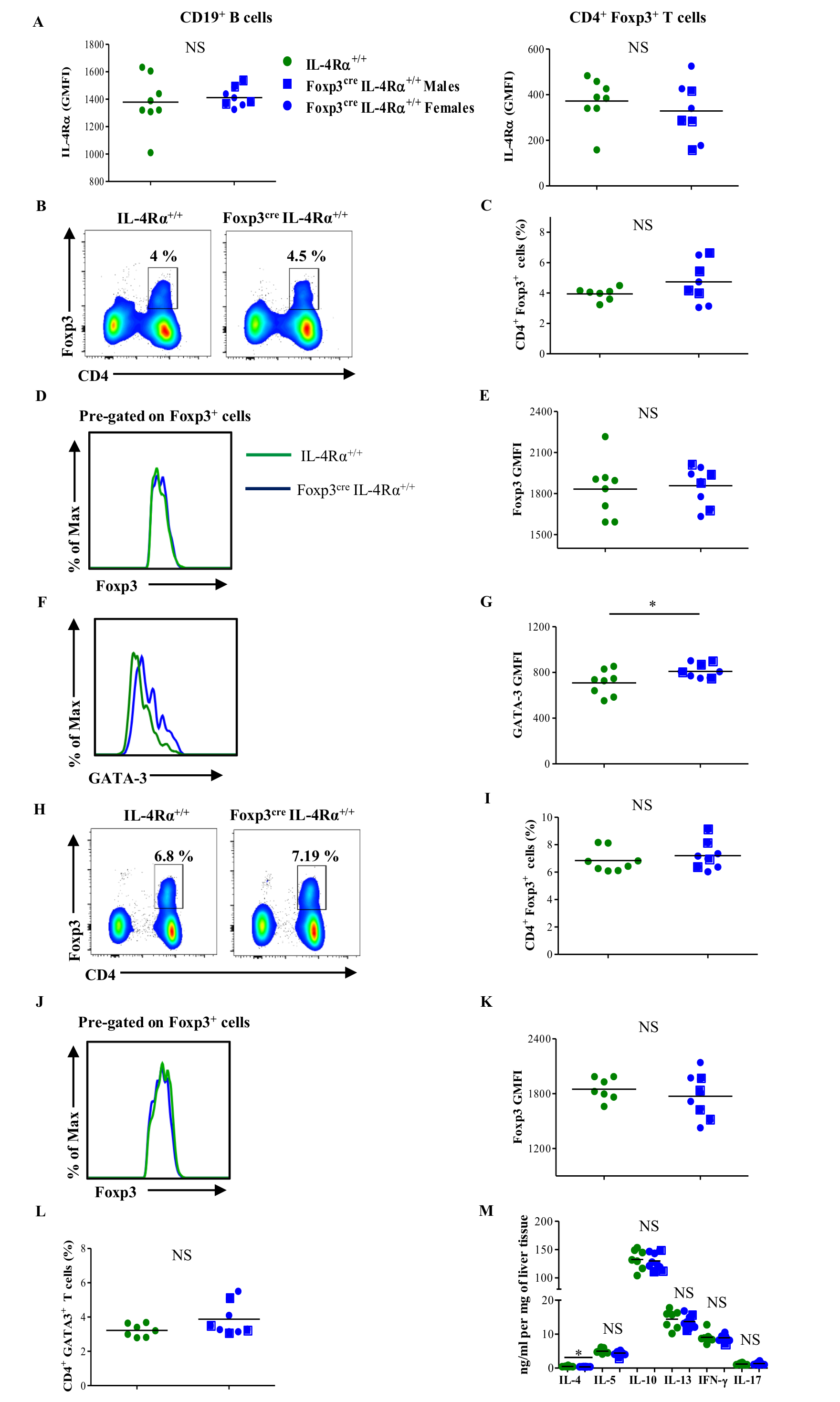

Supplement: S7 Fig — IL-4Rα+/+ and Foxp3cre IL-4Rα+/+ mice were infected with 100 Sm cercariae and euthanized 8 wk after, and Foxp3+ Treg cell compartment was analyzed in liver and MLN. (A) Flow cytometry analysis of IL-4Rα expression by CD19+ B cell and CD4+ Foxp3+ T cell in pooled spleen and MLN cells 8 wk post infection. (B) Representative flow cytometry of CD4+ Foxp3+ T cells in the liver. (C) Frequency of CD4+ Foxp3+ T cells from (A). (D) Representative histogram of Foxp3 expression by CD4+ Foxp3+ T cells in the liver. (E) Foxp3 GMFI in CD4+ Foxp3+ T cells from (D). (F) Representative histogram of GATA3 expression by CD4+ Foxp3+ T cells in the liver 8 wk post infection with the mean values summarized in (G). (H) Representative flow cytometry of CD4+ Foxp3+ T cells in the MLN. (I) Frequency of CD4+ Foxp3+ T cells from (H). (J) Representative histogram of Foxp3 expression by CD4+ Foxp3+ T cells in MLN. (K) Foxp3 GMFI in CD4+ Foxp3+ T cells from (J). (L) Frequency of CD4+ GATA3+ T cells in MLN 8 wk post infection. (M) Liver cytokine production 8 wk post infection. Livers from infected mice were homogenized, and the levels of the indicated cytokines were detected by ELISA and normalized to mg of liver tissue. Results are representative of two independent experiments with 6–8 mice/group. Data are expressed as mean ± S.E.M. NS, P > 0.05; * P < 0.05, ** P < 0.001, *** P < 0.0001 by two-tailed unpaired Student t test. Underlying data can be found in S1 Data. CD4, cluster of differentiation 4; CD19, cluster of differentiation 19; Cre, cyclic recombinase; Foxp3, forkhead box P3; GATA3, GATA binding protein 3; GMFI, geometric mean fluorescence intensity; IFN-γ, interferon gamma; IL-4, interleukin-4; IL-4Rα, interleukin-4 receptor alpha; IL-5, interleukin-5; IL-10, interleukin-10; IL-13, interleukin-13; IL-17, interleukin-17; MLN, mesenteric lymph node; NS, not significant; Treg, regulatory T. (TIF) [file pbio.2005850.s008.tif]

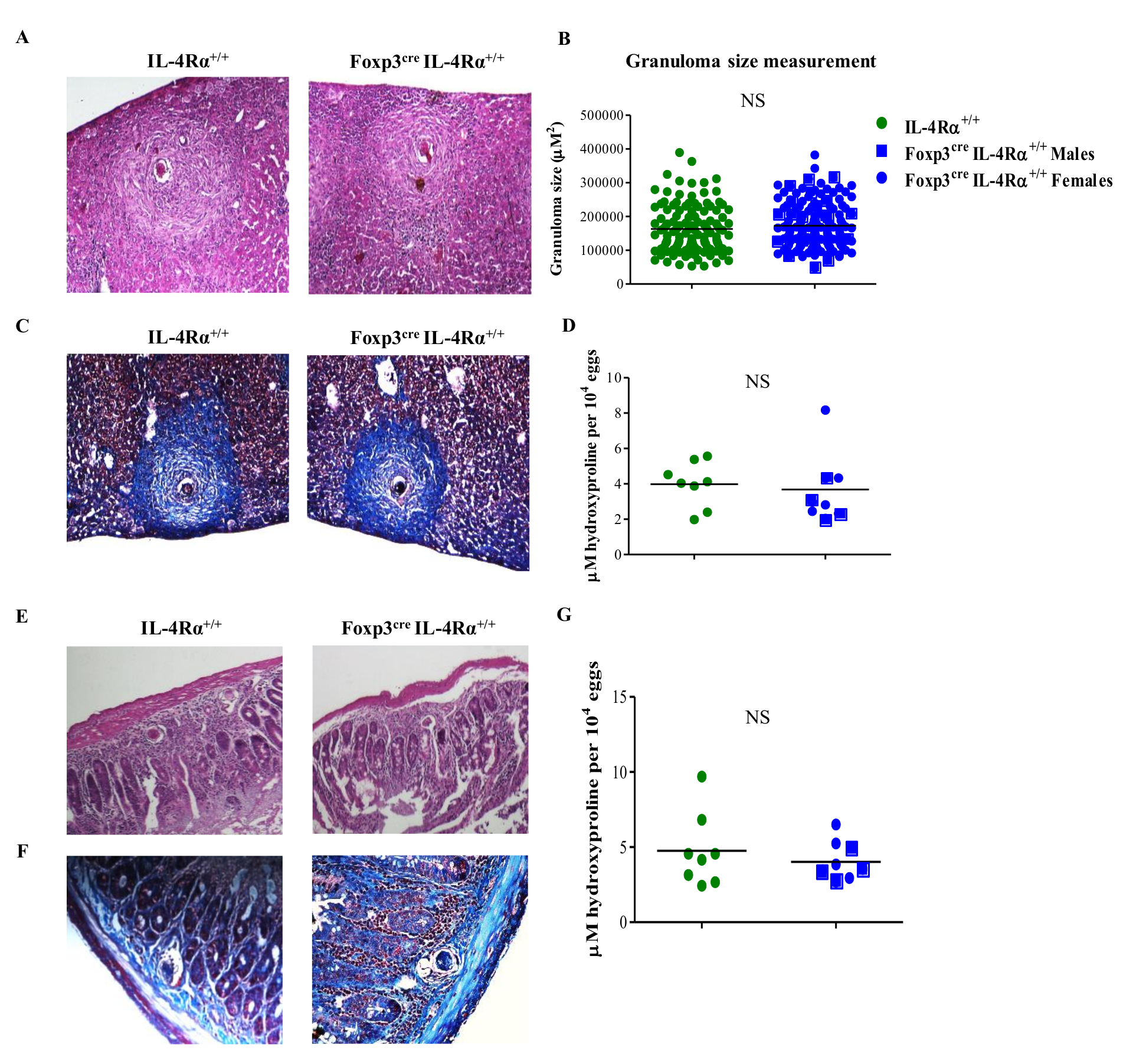

Supplement: S8 Fig — (A) Representative HE staining of liver sections from IL-4Rα−/Lox and Foxp3Cre IL-4Rα−/Lox mice infected with Sm for 8 wk (original magnification 100×). (B) Liver granuloma size. Granuloma size was determined from (A) by using a computerized morphometric analysis program (NIS elements by NIKON) by measuring 100 granulomas/group. (C) Representative CAB-stained liver sections from Sm-infected mice (original magnification 100×). (D) Liver hydroxyproline content measured by colorimetry 8 wk post infection. (E) Representative HE staining of gut sections from mice infected with Sm for 8 wk (original magnification 100×). (F) Representative CAB-stained gut sections 8 wk post infection (original magnification 100×). (G) Gut hydroxyproline content 8 wk post infection. Results are representative of two independent experiments with 6–8 mice/group. Data are expressed as mean ± S.E.M. NS, P > 0.05; * P < 0.05, ** P < 0.001, *** P < 0.0001 by two-tailed unpaired Student t test. Underlying data can be found in S1 Data. CAB, chromotrope aniline blue; Cre, cyclic recombinase; Foxp3, forkhead box P3; HE, hematoxylin–eosin; IL-4Rα, interleukin-4 receptor alpha; NS, not significant; Sm, S. mansoni. (TIF) [file pbio.2005850.s009.tif]

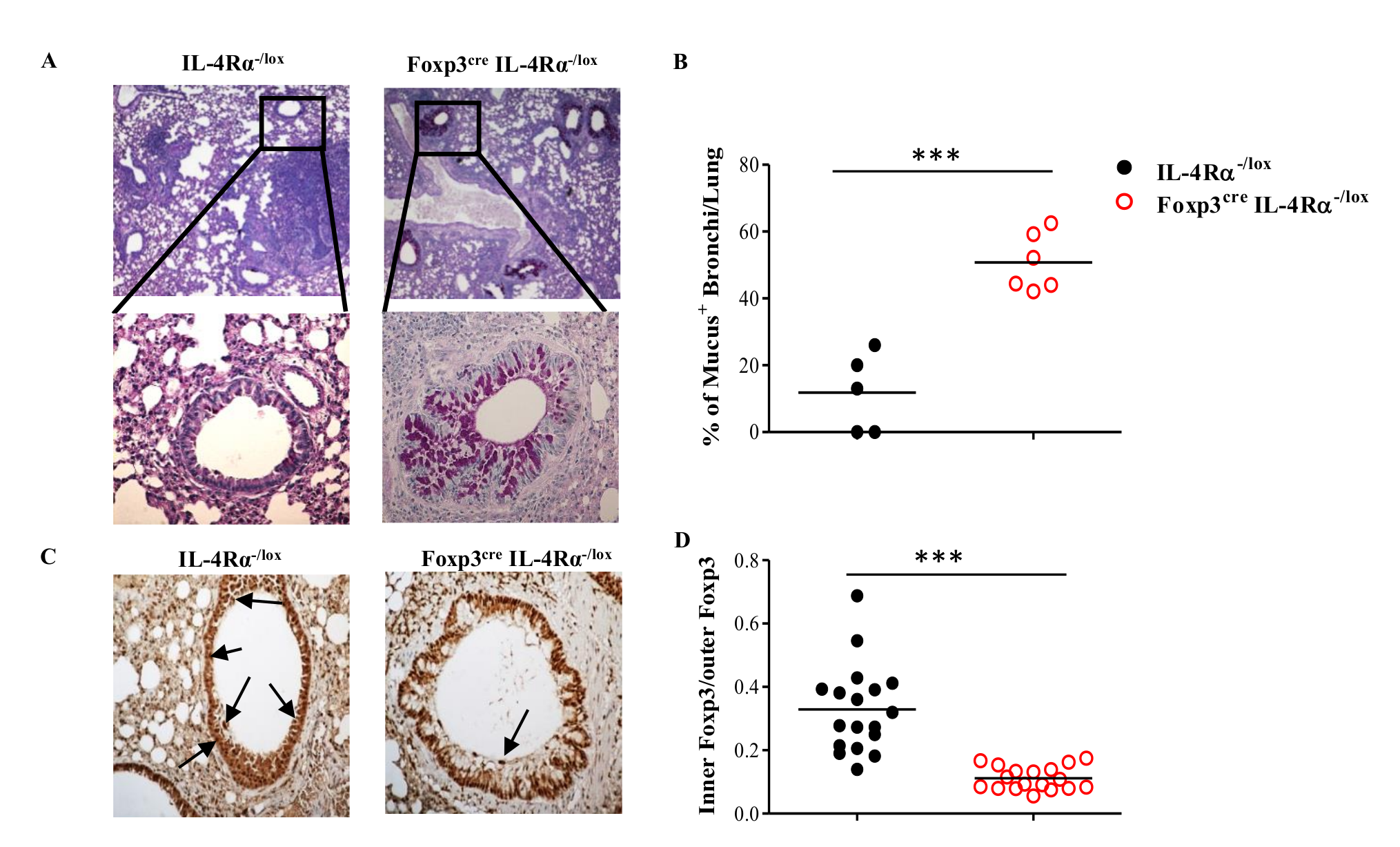

Supplement: S9 Fig — (A) Representative PAS staining of mucus-producing goblet cells, with lower (20×, top) and higher (200×) magnifications, in the lung tissues 9 d post infection of IL-4Rα−/lox and Foxp3cre IL-4Rα−/lox mice with 500 L3 larvae of Nb. (B) Quantification of PAS+ bronchi/lung/mouse. (C) Representative of Foxp3+ Treg cell infiltration within the lung alveoli 9 d post Nb infection (original magnification 200×). Thin arrows point to inner and outer Foxp3+ cells. (D) Ratio of inner to outer Foxp3+ Treg cells per alveoli. Results are representative of two independent experiments with 5–7 mice/group. Data are expressed as mean ± S.E.M. NS, P > 0.05; * P < 0.05, ** P < 0.001, *** P < 0.0001 by two-tailed unpaired Student t test. Underlying data can be found in S1 Data. Foxp3, forkhead box P3; IL-4Rα, interleukin-4 receptor alpha; Nb, N. brasiliensis; NS, not significant; PAS, periodic acid-Schiff reagent; Treg, regulatory T. (TIF) [file pbio.2005850.s010.tif]

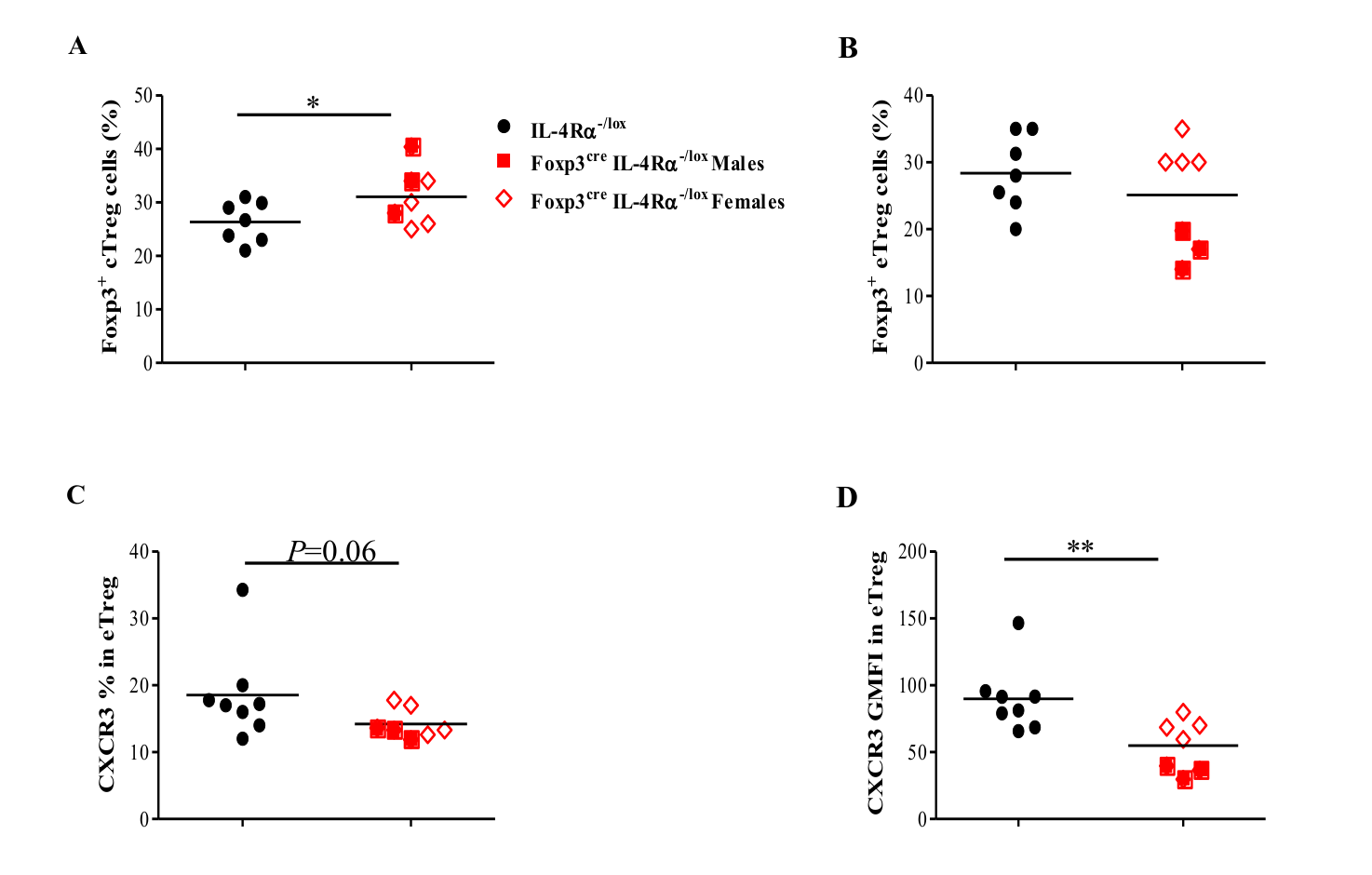

Supplement: S10 Fig — (A) Frequency of central CD4+ Foxp3+ Treg cells (left) and (B) effector CD4+ Foxp3+ Treg cells. (C) Frequency of CXCR3+ population within the effector CD4+ Foxp3+ Treg cells. (D) CXCR3 GMFI within the effector CD4+ Foxp3+ Treg cells. Results pooled from two independent experiments with 3–4 mice/group. Data are expressed as mean ± S.E.M. NS, P > 0.05; * P < 0.05, ** P < 0.001, *** P < 0.0001 by two-tailed unpaired Student t test. Underlying data can be found in S1 Data. cTreg, central Foxp3+ regulatory T; CXCR3, C-X-C motif chemokine receptor 3, eTreg, effector regulatory T; Foxp3, forkhead box P3; GMFI, geometric mean fluorescence intensity; IL-4Rα, interleukin-4 receptor alpha; MLN, mesenteric lymph node; NS, not significant; Treg, regulatory T. (TIF) [file pbio.2005850.s011.tif]
